# Raptor-Mediated Proteasomal Degradation of Deamidated 4E-BP2 Regulates Postnatal Neuronal Translation and NF-κB Activity

**DOI:** 10.1016/j.celrep.2019.11.023

**Published:** 2019-12-10

**Authors:** Stella Kouloulia, Erik I. Hallin, Konstanze Simbriger, Inês S. Amorim, Gilliard Lach, Theoklitos Amvrosiadis, Kleanthi Chalkiadaki, Agniete Kampaite, Vinh Tai Truong, Mehdi Hooshmandi, Seyed Mehdi Jafarnejad, Paul Skehel, Petri Kursula, Arkady Khoutorsky, Christos G. Gkogkas

**Affiliations:** 1 Centre for Discovery Brain Sciences and Patrick Wild Centre, University of Edinburgh, Edinburgh EH8 9XD, UK; 2 Department of Biomedicine, University of Bergen, Bergen N-5020, Norway; 3 Department of Anesthesia and Alan Edwards Centre for Research on Pain, McGill University, Montréal H3A 0G1, QC, Canada; 4 Centre for Cancer Research and Cell Biology, Queen’s University of Belfast, Belfast BT9 7AE, UK; 5 Faculty of Biochemistry and Molecular Medicine, University of Oulu, Oulu FI-90014, Finland; 6 Simons Initiative for the Developing Brain, University of Edinburgh, Edinburgh EH8 9XD, UK

**Keywords:** asparagine deamidation, translational control, postnatal brain, 4E-BP2, Raptor, CUL4B, proteasome, NF-κB, mTORC1

## Abstract

The translation initiation repressor 4E-BP2 is deamidated in the brain on asparagines N99/N102 during early postnatal brain development. This post-translational modification enhances 4E-BP2 association with Raptor, a central component of mTORC1 and alters the kinetics of excitatory synaptic transmission. We show that 4E-BP2 deamidation is neuron specific, occurs in the human brain, and changes 4E-BP2 subcellular localization, but not its disordered structure state. We demonstrate that deamidated 4E-BP2 is ubiquitinated more and degrades faster than the unmodified protein. We find that enhanced deamidated 4E-BP2 degradation is dependent on Raptor binding, concomitant with increased association with a Raptor-CUL4B E3 ubiquitin ligase complex. Deamidated 4E-BP2 stability is promoted by inhibiting mTORC1 or glutamate receptors. We further demonstrate that deamidated 4E-BP2 regulates the translation of a distinct pool of mRNAs linked to cerebral development, mitochondria, and NF-κB activity, and thus may be crucial for postnatal brain development in neurodevelopmental disorders, such as ASD.

## Highlights

• Deamidated 4E-BP2 occurs in neurons and is susceptible to ubiquitination/degradation
• mTORC1 or glutamate receptor inhibition stabilizes deamidated 4E-BP2
• A Raptor-CUL4B ubiquitin ligase complex binds to deamidated 4E-BP2
• Deamidated 4E-BP2 regulates postnatal brain translation and NF-κB activity

## Graphical Abstract

**Figure:**
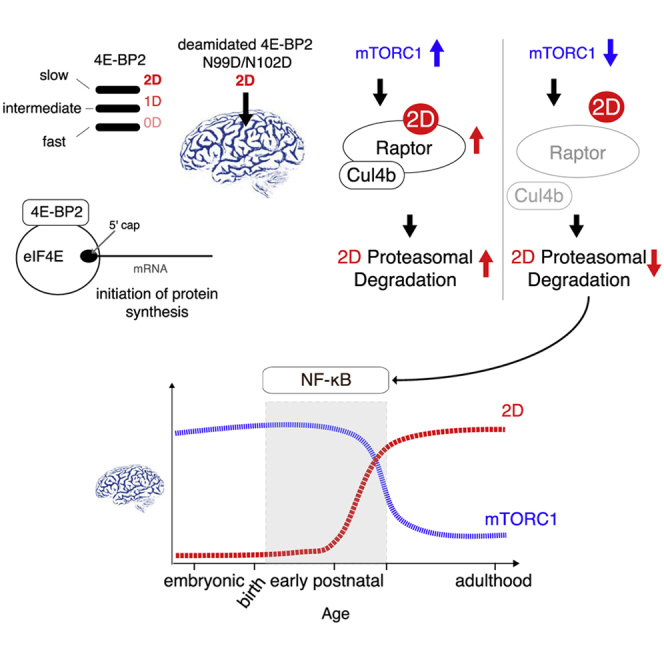

## Teaser

Kouloulia et al. demonstrate that, during early postnatal brain development, deamidation of the translation initiation factor 4E-BP2 renders it susceptible to ubiquitination and proteasomal degradation via enhanced binding to the Raptor-CUL4B complex. mTORC1 or glutamate receptor inhibition stabilizes deamidated 4E-BP2. Moreover, deamidated 4E-BP2 regulates the translation of specific mRNAs and NF-κB activity.

## Introduction

Since their discovery (Pause et al., 1994) as repressors of eukaryotic translation initiation factor 4E (eIF4E), eIF4E-binding proteins (4E-BPs) have been implicated in cancer progression (Petroulakis et al., 2009), innate immunity (Colina et al., 2008), circadian rhythms (Cao et al., 2013), learning and memory (Banko et al., 2005, Banko et al., 2007), and, more recently, autism spectrum disorders (ASDs) (Gkogkas et al., 2013). Cap-dependent translation initiation requires the binding of eIF4E to the 5′ end cap of the UTRs of mRNAs (Hinnebusch et al., 2016, Sonenberg and Hinnebusch, 2009). This event brings about the formation of the eIF4F translation initiation complex comprising, in addition to eIF4E, eIF4G (a scaffolding protein) and eIF4A (an mRNA helicase) (Gkogkas et al., 2010). 4E-BPs in their hypo-phosphorylated state inhibit eIF4F complex formation by competing with eIF4G for binding to the dorsal surface on eIF4E (Matsuo et al., 1997, Lukhele et al., 2013). Upon phosphorylation by the mammalian/mechanistic target of rapamycin complex 1 (mTORC1) kinase, 4E-BPs display a reduced affinity for eIF4E, leading to increased eIF4F complex formation, and thus translation initiates (Pause et al., 1994). This sophisticated signaling cascade, which constitutes a rate-limiting step for protein synthesis, has evolved to preferentially regulate the synthesis of specific proteins in different cell types and tissues, via mRNA translational control (Jung et al., 2014, Hinnebusch et al., 2016). There are three mammalian 4E-BPs (4E-BP1, 4E-BP2, and 4E-BP3), and 4E-BP2 is the paralog predominantly expressed in the mammalian brain (Banko et al., 2005, Tsukiyama-Kohara et al., 2001, Bidinosti et al., 2010b).

4E-BP2 is an intrinsically disordered protein (IDP), lacking tertiary structure, but upon phosphorylation-induced folding, the protein structure becomes stable (Bah et al., 2015). Phosphorylation of 4E-BP2 occurs in most tissues, including in the brain. However, during postnatal brain development, 4E-BP2 phosphorylation decreases (Banko et al., 2005, Bidinosti et al., 2010b), coinciding with an overall decrease in mTORC1 signaling (Bidinosti et al., 2010b). 4E-BP2 phosphorylation is barely detectable in the adult brain (Banko et al., 2005, Bidinosti et al., 2010b). Notably, in early postnatal brain development and exclusively in brain tissue, 4E-BP2 undergoes post-translational deamidation (persisting into adulthood) on asparagines N99 and N102, which are converted to a mixture of aspartates and isoaspartates (Bidinosti et al., 2010b). Because isoaspartates can destabilize proteins, the enzyme l-isoaspartyl methyltransferase (PIMT), which is highly active in the brain, catalyzes the conversion of isoaspartates to aspartates (via the intermediate product succinimide), and 4E-BP2 was shown to be a PIMT substrate (Bidinosti et al., 2010a). Asparagine deamidation is not catalyzed by enzymes, but it can occur spontaneously and is accelerated by alkaline pH (Robinson, 2002). The only two known examples of enzymatic asparagine deamidation are linked to herpes simplex virus (HSV). First, asparagine deamidation of the pattern recognition receptor RIG-I (retinoic acid-induced gene I) by cellular phosphoribosylformyglycinamide synthase (PFAS) during HSV infection serves as a mechanism to avoid antiviral cytokine production (He et al., 2015). Second, the HSV tegument protein UL37 deamidates cellular cyclic guanosine monophosphate (GMP)-AMP synthase (cGAS) to attenuate innate immunity (Zhang et al., 2018).

Deamidated 4E-BP2 was shown to regulate the kinetics of excitatory synaptic transmission in early postnatal brain development (Bidinosti et al., 2010b), suggesting that it may be important for synaptic function during that crucial developmental period. N99/N102 deamidation decreases the capacity of 4E-BP2 to compete with eIF4G and inhibit eIF4F complex formation, and it also increases its binding to the mTORC1 protein Raptor (Bidinosti et al., 2010b). The significance of enhanced Raptor binding to deamidated 4E-BP2 remains unclear. In addition, and given the pervasive role of 4E-BP2 in regulating brain function during development and adulthood (Gkogkas et al., 2013, Cao et al., 2013, Bidinosti et al., 2010b, Banko et al., 2005), it is crucial to elucidate the downstream effects of deamidated 4E-BP2 in regulating the translational landscape of mRNAs in the brain.

Here, we demonstrate that 4E-BP2 deamidation occurs in neurons but not glial cells and is also detected in the human brain. Constitutively deamidated 4E-BP2 protein is less stable than wild type (WT), and mTORC1 or glutamate receptor inhibition selectively promotes the accumulation of deamidated 4E-BP2 but not of unmodified protein. We reveal that the susceptibility of deamidated 4E-BP2 to proteasomal degradation depends on increased Raptor binding and Cullin 4B (CUL4B) protein abundance, leading to the enhanced formation of a Raptor- CUL4B E3 ligase complex. Using unbiased translatome mapping, we demonstrate that deamidated 4E-BP2 preferentially regulates the synthesis of a distinct pool of mRNAs linked to neuronal development, proliferation, glutathione and oxidoreductase activity, mitochondrial function, and nuclear factor κ-light-chain enhancer of activated B cells (NF-κB) activity. Moreover, the overexpression of deamidated 4E-BP2 strongly inhibits NF-κB activity. These data describe a previously unidentified brain-specific translational control mechanism during early postnatal brain development (persisting into adulthood), which could be crucial for neurodevelopmental disorders.

## Results

### Postnatal 4E-BP2 Deamidation Is Neuron Specific, but It Does Not Alter Its Intrinsically Disordered State

**Figure 1:**
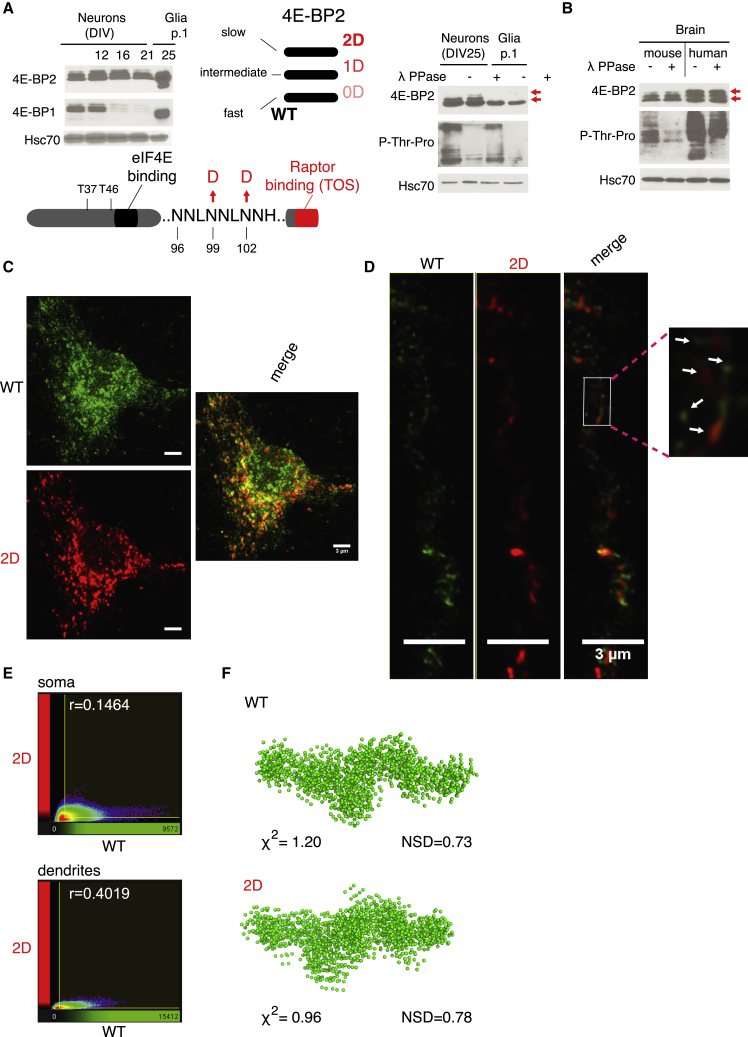
Postnatal 4E-BP2 Deamidation Is Neuron Specific, Affects Protein Subcellular Localization, but Does Not Alter Its Intrinsically Disordered State (A) Left: representative immunoblots of lysates from different days *in vitro* (DIV) neurons cultured in the presence of 1 μM Ara-C or glial cells re-plated after trypsinization of neuronal cultures, probed with antisera against the indicated proteins; n = 3. Right: representative immunoblots of lysates from DIV25 neurons or passage 1 (p.1) glial cells treated with λ-phosphatase (λ-PPase). Hsc70 is a loading control; n = 2. Middle: schematic diagram of the SDS-PAGE migration pattern of 4E-BP2 in brain tissue showing 3 distinct forms: 0D (no deamidation), 1D (N99D or N102D), and 2D (N99D/N102D). Bottom: schematic of the major domains in 4E-BP2 around the deamidation site, mTOR phosphorylation sites (T37/46), eIF4E binding site, and Raptor-binding domain (containing the TOS [TOR signaling] motif). (B) Immunoblotting of lysates prepared from mouse brain and post-mortem human brain treated with λ-phosphatase (λ-PPase) (see Table S1); n = 2. For (A) and (B), red arrows indicate the position of the slow migrating deamidated forms of 4E-BP2 on blots. Representative confocal microscopy images at 488 (green) or 680 (red) nm and a merged image are shown. (C and D) Soma (C) and dendrites (D) from dissociated DIV16 cortical mouse neurons co-transfected with WT (FLAG-tag) and 2D (HA-tag) 4E-BP2 and probed first with antisera against FLAG- or HA-tags, followed by secondary antibodies (conjugated to WT, green, Alexa Fluor 488; 2D, red, DyLight 680). Scale bars (3 μm) and arrows marking distinct WT or 2D fluorescent puncta are shown in white; n = 8. (E) Imaris-generated 2D histograms showing the quantification of fluorescent intensity measured images from (C, soma) and (D, dendrites), displaying the Pearson correlation coefficient of the colocalized volume of the red (2D) channel over the green (WT) channel. (F) Ten superimposed DAMMIN models, generated using the SAXS data from the highest protein concentration with the average χ^2^ score and normalized spatial discrepancy (NSD) for WT and 2D recombinant proteins. See also Figures S1, S2, and S3 and Tables S1 and S2.

To investigate the role of constitutively deamidated (asparagine→aspartic acid; D99/D102) as compared to non-deamidated, unmodified 4E-BP2 (N99/N102) in the brain, we cultured cortical neurons isolated from embryonic day (E)16–E18 mouse embryo cortices until day *in vitro* 25 (DIV25), when synapses are known to form in culture. Neurons were cultured in the presence of the mitotic inhibitor Ara-C (cytosine arabinose), which limits astrocyte proliferation. The expression of 4E-BP1 decreased significantly by DIV25 in neurons, as compared to glia, while 4E-BP2 expression remained stable (Figures 1A, left, and S1A). In addition to the ∼17-kDa band corresponding to non-deamidated 4E-BP2, we also observed 2 slower migrating bands recognized by the 4E-BP2 antibody in SDS-PAGE from cortical neurons at DIV12 (Figure 1A, left), which were previously shown to correspond to single and double deamidated 4E-BP2 (Bidinosti et al., 2010b; Figure 1A, middle graphic). To determine whether 4E-BP2 deamidation occurs in neurons or in glia, we used trypsin to dissociate cells from culture dishes at DIV10. By re-plating glial cells (passage 1 [p.1]), we effectively removed all neuronal cells that failed to re-attach. Following immunoblotting of glial lysates with the 4E-BP2 antibody, we detected only non-deamidated 4E-BP2 species (<17 kDa) (Figure 1A, right), thus revealing that mouse brain-derived glia express only non-deamidated 4E-BP2, displaying a faster migration pattern compared to neuronal constitutively deamidated 4E-BP2. Moreover, treatment with λ-phosphatase did not affect the migration pattern of neuronal DIV25 4E-BP2, in accordance with previous findings (Bidinosti et al., 2010b), but it did reduce overall phosphorylation in neurons and in p.1 glia, as detected by phospho-serine/threonine antisera (Figure 1A, right). Notably, 4E-BP1 is highly expressed in glia, as compared to DIV25 neurons (Figures 1A, left, and S1A). Because these experiments were carried out in mouse brain-derived cells, we sought to identify whether 4E-BP2 deamidation also occurs in the human brain. Immunoblotting of post-mortem human brain tissue lysates with the 4E-BP2 antibody showed 2 slower migrating bands >17 kDa, which are resistant to λ-phosphatase treatment, similar to mouse brain (Figures 1B and S1B). Thus, these data suggest that 4E-BP2 deamidation is neuron specific in the mouse brain and also takes place in the adult human brain.

To further study the differences between non-deamidated and constitutively deamidated 4E-BP2 in neurons, we co-transfected DIV4 cortical neurons with plasmids expressing N-terminally tagged non-deamidated (WT; FLAG-tag) and constitutively deamidated (2D; hemagglutinin [HA]-tag) 4E-BP2 (Figures 1C and 1D). 4E-BP2 is implicated in cytoplasmic translation initiation, and using immunofluorescence and confocal microscopy, we detected punctate WT and 2D staining in the neuronal cell body and the nucleus (Figure 1C), but also in dendrites in DIV16 cultured neurons (Figure 1D), which we further validated by microtubule-associated protein 2 (MAP2) co-staining of neuronal dendrites (Figure S2A). Whereas no significant differences were observed in the gross spatial expression of WT and 2D (apart from a decrease in 2D nuclear staining), there was very little overlap between the WT and 2D puncta, as measured by the co-localization of WT and 2D fluorescent signals, both in soma (r = 0.1464) and in dendrites (r = 0.4019) (Figure 1E).

Asparagine deamidation may alter protein structure (Robinson, 2002). Thus, we set out to determine the structure of deamidated 4E-BP2. 4E-BP2 is an IDP (Bah et al., 2015), and because it lacks ordered 3-dimensional structure, we could not use crystallography. To elucidate WT and 2D structures, we carried out synchrotron radiation circular dichroism (SRCD), small-angle X-ray scattering (SAXS), and nuclear magnetic resonance (NMR) spectroscopy of full-length recombinant 4E-BP2 (WT or 2D) expressed in *Escherichia coli* and purified in the monomeric state. First, SRCD spectra for WT and 2D show that both proteins contain random coils, as evidenced by the negative peak located near 199 nm (Figure S2B). Second, SAXS scatter profiles for WT and 2D overlap closely, showing featureless profiles for both proteins (Figure S2C). Kratky plots for both WT and 2D confirm their unfolded status (Figure S2D), in accordance with the SRCD results, suggesting that the protein is flexible and dominated by random coil structure. The size of WT and 2D proteins appears to depend on concentration (Figure S2E), which could suggest the presence of a dimer/monomer mixture. The molecular mass determined for 4E-BP2 using size exclusion chromatography-multi-angle light scattering (SEC-MALS) was 15.2 kDa, which is closer to a monomeric state. The *ab initio* models generated by DAMMIN and GASBOR fit the experimental SAXS data well and show a similar elongated shape for both WT and 2D (Figure 1F). Third, NMR spectra show peaks located in a narrow region, indicative of an IDP (Figure S3). The majority of the peaks in WT protein spectra overlap with 2D, suggesting that they share a similar structure, with only minor differences in several residues (Figure S3).

These data reveal that 4E-BP2 deamidation is neuron specific, occurs in the human brain, affects protein subcellular localization, and does not alter the 4E-BP2 IDP state.

### Accelerated Proteasomal Degradation and Increased Ubiquitination of Deamidated 4E-BP2

**Figure 2:**
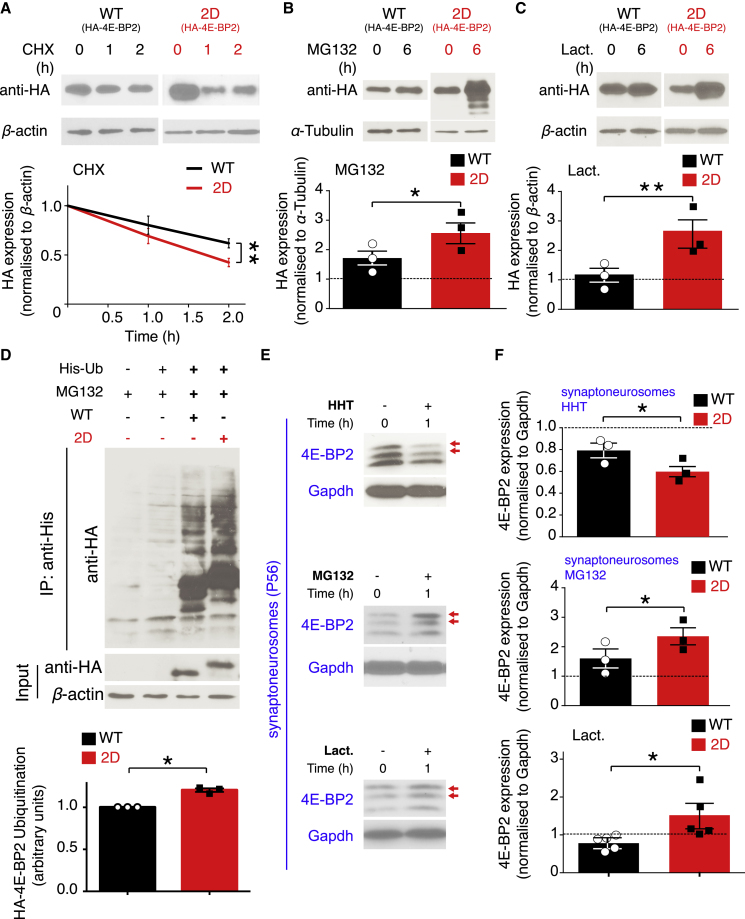
Accelerated Proteasomal Degradation and Increased Ubiquitination of Deamidated 4E-BP2 (A–C) Protein stability assays in HEK293H cells transfected with WT or 2D HA-tagged forms of 4E-BP2. Top: representative immunoblots of lysates treated with (A) cycloheximide 100 μg/mL; 0, 1, and 2 h (CHX); (B) MG132 20 μM; 0 and 6 h; and (C) lactacystin (Lact.) 5 μM; 0 and 6 h, probed with antisera against the indicated proteins. Bottom: quantification of HA expression normalized against the loading control (β-actin, A and C, or α*-*tubulin, B) for the indicated time points. Data are shown as means ± SEMs (error bars); n = 3 per construct. The intensity of the 0-h band is set as 1 (dotted line on graph). Two-way ANOVA; Bonferroni’s post hoc; ^∗^p < 0.05, ^∗∗^p < 0.01. (D) Top: ubiquitination assay in HEK293H cells. Representative immunoblots from transfected cells with His-ubiquitin and WT or 2D HA-tagged 4E-BP2, treated with MG132 20 μM for 6 h, from His-immunoprecipitated (IP) or input lysates probed with antisera against the indicated proteins. β-Actin serves as a loading control. Bottom: quantification of ubiquitination assay data; n = 3, Student’s t test, ^∗^p < 0.05. (E) Protein stability assays in P56 brain isolated synaptoneurosomes. Representative immunoblots of lysates treated for 1 h with HHT 2 μg/mL, MG132 20 μM, Lact. 10 μM; probed with antisera against the indicated proteins. Glyceraldehyde 3-phosphate dehydrogenase (GAPDH) is the loading control. (F) Quantification of endogenous 4E-BP2 expression in synaptoneurosomes from (E), normalized to the loading control. Data are shown as means ± SEMs (error bars); n = 3 for all groups apart from lactacystin (n = 5). The intensity of the 0-h band is set as 1 (dotted line on graph); two-way ANOVA; Bonferroni’s post hoc; ^∗^p < 0.05. Red arrows mark the slower migration of single or double deamidated 4E-BP2 protein. See also Figure S4 and Table S2.

Since asparagine deamidation may alter protein stability (Robinson, 2002), we tested the stability of WT and 2D 4E-BP2 by transfecting HA-tagged constructs into HEK293H cells. Treatment with cycloheximide (CHX), an inhibitor of the elongation phase of protein synthesis, for 1 or 2 h, led to the rapid degradation of 2D compared to WT at 2 h, as evidenced by HA expression measured with immunoblotting (Figure 2A). Conversely, inhibition of the proteasome either with MG132 (Figure 2B) or lactacystin for 6 h (Figure 2C) led to a significant accumulation of HA-tagged 2D compared to WT (Figures 2B and 2C). Endogenous 4E-BP2 in HEK293H cells or a transfected N99A/N102A (2A) mutant follow a stability pattern similar to transfected WT (Figures S4A–S4D). Changes in protein stability due to proteasomal degradation are often preceded by changes in the ubiquitination status of a protein. Therefore, we used histidine-tagged ubiquitin (His-Ub) immunoprecipitation in HEK293H cells transfected with WT or 2D and immunoblotting to detect ubiquitinated protein species (Figure 2D). We detected significantly more HA-2D in His-Ub immunoprecipitates than HA-WT (Figure 2D), thus demonstrating that the N99D/N102D mutation engenders increased polyubiquitination, which leads to enhanced proteasomal degradation.

We then proceeded to examine whether we could recapitulate the short half-life of 2D in neurons. In lysates from cultured DIV25 cortical neurons treated with CHX, lactacystin, or MG132, we detected changes only in 4E-BP2 stability in the MG132-treated samples (Figure S4E). Inhibition of the proteasome for 9 h with MG132 led to a significantly higher accumulation of deamidated (∼1.5-fold; p = 0.009) compared to non-deamidated 4E-BP2, as evidenced by the increased intensity of the slower migrating bands detected by the 4E-BP2 antibody with immunoblotting (Figure S4E). These data suggest that 4E-BP2 could be more stable in neurons compared to HEK293H cells. To further test this hypothesis, we treated neurons with a different elongation inhibitor, homoharringtonine (HHT); however, we did not observe any decrease in 4E-BP2 expression after 2 or 9 h (Figure S4E).

Dendritic expression of 4E-BP2 (Figures 1C and 1D) led us to hypothesize that 4E-BP2 stability could be altered in synaptic fractions. 4E-BP2 (non-deamidated and deamidated) is expressed in synaptoneurosomal fractions isolated from P56 mouse brain, along with the expression of synaptic proteins (PSD95, synaptophysin) and the depletion of glial fibrillary acidic protein (GFAP) and nuclear (histone H3) marker protein (Figure S4F). In synaptoneurosomes, HHT treatment induced degradation of deamidated 4E-BP2 significantly more than non-deamidated (Figures 2E and 2F). Conversely, the treatment of synaptoneurosomes with MG132 or lactacystin led to a pronounced increase in deamidated compared to non-deamidated 4E-BP2 expression (Figures 2E and 2F). These data suggest that 4E-BP2 deamidation reduces 4E-BP2 stability by increasing 2D ubiquitination and proteasomal degradation and that this mechanism is present in synaptic fractions.

### mTOR or Glutamate Receptor Inhibition Promotes Accumulation of Deamidated 4E-BP2

**Figure 3:**
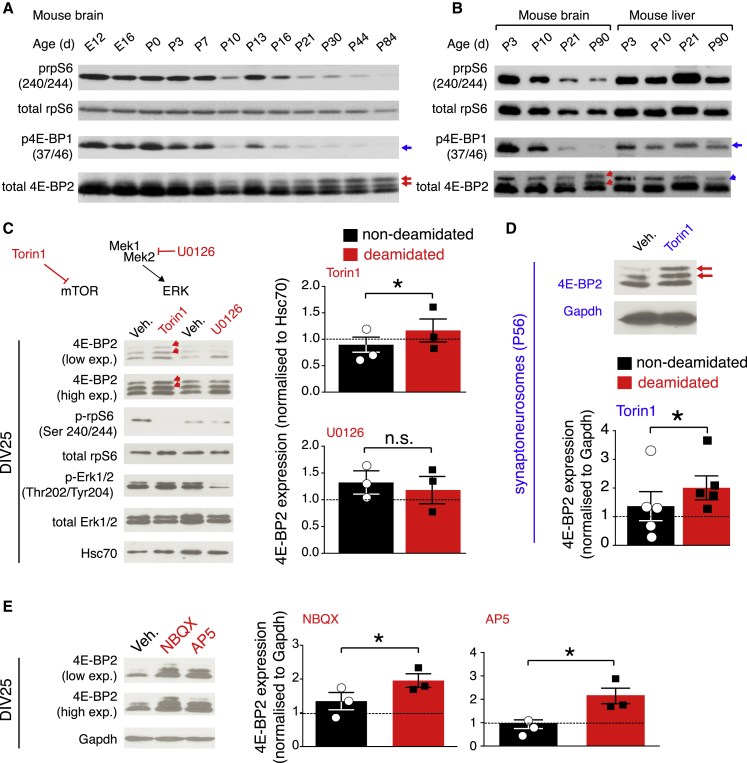
mTOR, AMPAR, or NMDAR Inhibition Promotes the Accumulation of Deamidated 4E-BP2 (A) mTORC1 and 4E-BP2 deamidation in the brain. Immunoblotting of mouse forebrain lysates collected at different ages and probed with antisera against the indicated proteins. (B) mTORC1 and 4E-BP2 deamidation in the brain versus the liver. (C) Top left: diagrammatic depiction of pathways inhibited by Torin 1 and U0126 inhibitors. Bottom left: representative immunoblots from DIV25 neuron lysates treated with vehicle or 250 nM Torin 1, or vehicle or 20 μM U0126 for 9 h, probed with antisera against the indicated proteins; Hsc70 is a loading control. Right: quantification of immunoblots; 4E-BP2 expression normalized to control is shown for WT and deamidated (2D) 4E-BP2. (D) Top: representative immunoblots from P56 mouse brain-extracted synaptoneurosomes treated with vehicle or 250 nM Torin 1 for 1 h, probed with antisera against the indicated proteins. Bottom: quantification of immunoblots; 4E-BP2 expression normalized to control is shown for WT and deamidated (2D) 4E-BP2. (E) Representative immunoblots (left) and quantification of 4E-BP2 expression normalized to control (right) from DIV25 neuron lysates treated with vehicle, 10 μM NBQX, or 50 μM AP5 for 9 h, probed with antisera against the indicated proteins. GAPDH is the loading control. For (A) and (B), the blue arrows show the phosphorylation of 4E-BP2, while the red arrows mark the slower migrating forms of deamidated (single or double) 4E-BP2 protein. For (C)–(E), the data are shown as means ± SEMs (error bars). For (C) and (E), n = 3 per condition. For (D), n = 5 per condition. The intensity of the vehicle band is set as 1 (dotted line on graph). Two-way ANOVA; Bonferroni’s post hoc; ^∗^p < 0.05, ^∗∗^p < 0.01. See also Figures S4 and S5 and Table S2.

mTORC1 is a major signaling pathway regulating translation initiation. mTORC1 activity is significantly downregulated during early postnatal brain development (postnatal day [P]10–P21), overlapping with the period when 4E-BP2 deamidation arises (Figure 3A). Immunoblotting of forebrain lysates prepared from different ages (E12–P84) reveals a marked decrease in the phosphorylation of the major downstream translation initiation effectors of mTORC1 (4E-BP2 and rpS6), concomitant with an increase in the expression of deamidated 4E-BP2 (Figure 3A). This result was recapitulated in cerebellar tissue (data not shown), suggesting that this is a brain-wide phenomenon occurring during early postnatal development and is in accordance with previous findings (Bidinosti et al., 2010b, Li et al., 2016). We did not detect this postnatal decrease in mTORC1 signaling in liver tissue (Figure 3B) or in cultured mouse glia (Figure S4G), which strongly suggests that this is a neuron-specific phenomenon.

To establish a causal relation between postnatal decrease in mTORC1 signaling and the stability of 4E-BP2, we treated cultured mouse cortical neurons with a selective active-site mTOR inhibitor, Torin 1 (Thoreen et al., 2009; Figure 3C). Torin 1 treatment led to a significant increase in deamidated protein amounts compared to non-deamidated at 9 h (Figure 3C) or 48 h (Figures S4H and S4I). The treatment of neurons for the same duration with the MEK inhibitor U0126 upstream of ERK (Favata et al., 1998) did not affect the stability of either 4E-BP2 form at 9 h (Figure 3C) or 48 h (Figures S4H and S4I). We did not detect any changes in 4E-BP2 protein stability in glia following Torin 1 treatment, further suggesting that this is a neuron-specific mechanism (Figure S5A). Furthermore, Torin 1-treated synaptoneurosomes analyzed with immunoblotting reveal a significant increase in deamidated protein expression compared to non-deamidated (Figure 3D). Likewise, treatment of synaptoneurosomes with 20 nM rapamycin led to a significant accumulation of deamidated protein versus non-deamidated protein (Figure S5B). mTORC1 promotes the activity of the proteasome (Zhao et al., 2015, Zhao et al., 2016). Along these lines, in Torin 1 or rapamycin-treated synaptoneurosomes, we observed a marked decrease in 20S proteasome activity (Figure S5C).

These data suggest that mTORC1 inhibition selectively promotes the neuronal stability of deamidated 4E-BP2 by inhibiting the activity of the proteasome.

mTORC1 activity is strongly regulated by α-amino-3-hydroxy-5-methyl-4-isoxazolepropionic acid receptor (AMPAR) or *N*-methyl-d-aspartate receptor (NMDAR) activity (Gong et al., 2006). Thus, we proceeded to examine whether the inhibition of AMPARs or NMDARs would affect 4E-BP2 protein stability. Treating DIV25 neurons with the AMPAR antagonist 2,3-dihydroxy-6-nitro-7-sulfamoyl-benzo[f]quinoxaline (NBQX) or the NMDAR antagonist AP5 for 9 h induced the accumulation of deamidated compared to non-deamidated 4E-BP2, similarly to Torin 1 treatment (Figure 3E). Thus, AMPAR or NMDAR inhibition promotes the accumulation of deamidated compared to non-deamidated 4E-BP2. This could occur either via inhibiting mTORC1 or by acting directly on the proteasome.

Therefore, these data describe a neuron-specific mechanism whereby mTORC1 or glutamate receptor inhibition promotes the accumulation of deamidated 4E-BP2, most likely by inhibiting the activity of the proteasome.

### Increased Binding to Raptor and CUL4B Boosts 4E-BP2 Proteasomal Degradation

**Figure 4:**
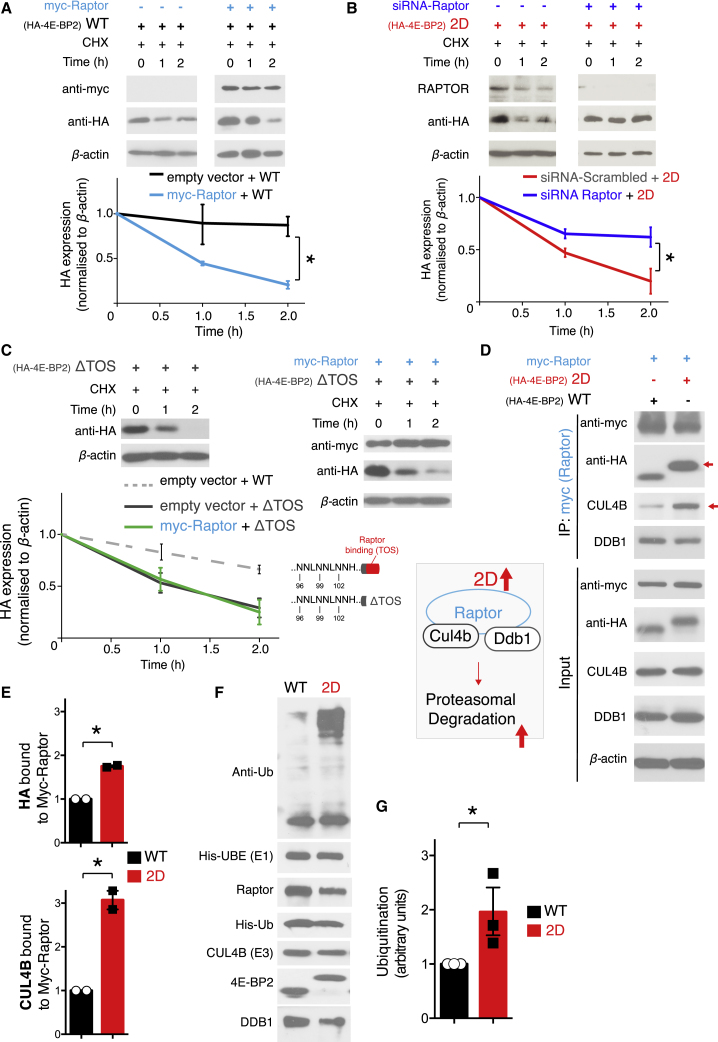
Increased Binding to Raptor and CUL4B Boosts 4E-BP2 Proteasomal Degradation (A–C) Top: representative immunoblots from HEK293H lysates co-transfected with myc-Raptor (A). WT HA-tagged 4E-BP2 and empty vector or myc-raptor (B) co-transfected with siRNA (scrambled or against *RAPTOR)* and 2D HA-tagged 4E-BP2, or (C) HA-4E-BP2 ΔTOS with empty vector or myc-Raptor + HA-4E-BP2 ΔTOS. Bottom: quantification of HA expression (corresponding to WT or 2D) measured by immunoblotting, normalized to β*-*actin. In (C, bottom), a depiction of 4E-BP2 domains is shown to highlight the C-terminal deletion of the TOS motif (ΔTOS). (D) Representative immunoblots from HEK293H immunoprecipitates (IP; top, with anti-myc antisera) and whole lysates (bottom), co-transfected with myc-Raptor and WT or 2D HA-tagged 4E-BP2 and probed with the antisera against the indicated proteins; β*-*actin is the loading control. The red arrow shows increased expression in IP. Right: depiction of the Raptor-CUL4B-DDB1-2D complex. (E) Quantification of data in (D) for HA expression and CUL4B bound to myc-Raptor, Student’s t test (n = 2),^∗^p < 0.05. (F) *In vitro* ubiquitination assay of purified GST-4E-BP2 WT or 2D. The reactions were performed in the presence of purified CUL4B, Raptor, DDB1, His-ubiquitin, UBE2L3, and UBE1 proteins and probed with the antisera against the indicated proteins. GST, glutathione S-transferase. (G) Quantification of ubiquitination data in (E); Student’s t test (n = 3); ^∗^p < 0.05. For (A)–(C), all of the experiments were carried out in the presence of 100 μg/mL cycloheximide (CHX) for 0, 1, or 2 h; β*-*actin is the loading control. The data are shown as means ± SEMs (error bars); n = 3 per condition; two-way ANOVA; Bonferroni’s post hoc; ^∗∗^p < 0.01, ^∗∗^p < 0.001. See also Figure S5 and Table S2.

mTORC1 activation leads to the increased binding of its major subunit, Raptor, to 4E-BPs (Schalm et al., 2003). We demonstrated that mTORC1 inhibition, which decreases Raptor-4E-BP binding, stimulates deamidated 4E-BP2 accumulation (Figure 3). We thus hypothesized that the increased affinity of deamidated 4E-BP2 for the mTORC1 protein subunit Raptor (Bidinosti et al., 2010b) could be responsible for enhanced deamidated 4E-BP2 proteasomal degradation. To test this hypothesis, first, we co-transfected HEK293H cells with WT HA-tagged 4E-BP2 along with a plasmid encoding myc-tagged full-length Raptor (Figure 4A). CHX treatment for 1 or 2 h led to a significant decrease (p < 0.01) in the expression of HA-tagged WT 4E-BP2 co-transfected with myc-Raptor, as compared to WT co-transfected with empty vector (Figure 4A). Thus, Raptor co-transfection reduces the stability of WT 4E-BP2, making it behave similarly to 2D (Figure 2A). However, myc-Raptor overexpression dramatically reduced 2D 4E-BP2 expression (∼70% reduction), and after 2 h of CHX treatment, the protein does not degrade further, suggesting that it has reached a plateau (Figure S5D, red dotted line). Second, if an increase in Raptor binding (mimicked by Raptor overexpression in this experiment) is causal for the decreased stability of 2D, then reducing Raptor protein amounts should reverse this phenotype. Co-transfection of a small interfering RNA (siRNA) against human *RPTOR* with HA-tagged 2D decreases RAPTOR expression and increases the stability of 2D after 2 h of CHX treatment, as compared to co-transfection with a scrambled siRNA (Figure 4B). Moreover, co-transfection of a deletion construct of WT 4E-BP2 lacking the Raptor-binding domain (WT-ΔTOS) with myc-Raptor in HEK293H cells did not alter 4E-BP2-ΔTOS stability (Figure 4C), suggesting that 4E-BP2 binding to Raptor is required to induce protein instability. WT-ΔTOS degrades faster than full-length WT 4E-BP2, most likely because of its shorter length. Likewise, 2D-ΔTOS degrades faster than full-length 2D, but WT-ΔTOS and 2D-ΔTOS have similar degradation rates (Figure S5E). Third, to identify the underlying mechanism by which Raptor facilitates increased proteasomal degradation of 2D, we examined a ubiquitin E3 ligase complex comprising CUL4B and DNA damage-binding protein 1 (DDB1), because this complex binds Raptor (Ghosh et al., 2008). We co-transfected HEK293H cells with myc-Raptor and HA-tagged WT or 2D and processed lysates with myc immunoprecipitation to isolate Raptor-bound proteins (Figure 4D). By subjecting immunoprecipitates to immunoblotting, we detected significantly more HA-tagged 2D bound to myc-Raptor, as compared to HA-tagged WT (Figure 4E). Further probing of these samples with CUL4B and DDB1 antisera revealed increased binding of CUL4B to myc-Raptor in the presence of 2D, when compared to WT (Figures 4D and 4E). DDB1 binding was not changed between WT and 2D samples, which is in accordance with Ghosh et al. (2008). We did not observe any differences in the protein expression of myc-Raptor, CUL4B, or DDB1 between WT and 2D in input lysates (Figure 4D). To further elucidate the connection of CUL4B to 2D 4E-BP2 stability, we knocked down endogenous *CUL4B* in HEK cells using siRNAs and measured the stability of co-transfected HA-tagged 2D 4E-BP2 (Figure S5F). We observed that the reduced expression of endogenous CUL4B correlates with the increased stability of 2D following CHX treatment (Figure S5D). In addition, recombinant 2D is significantly more ubiquitinated in a CUL4B-dependent *in vitro* ubiquitination assay, as compared to WT (Figures 4F, 4G, and S5G). In conclusion, these data strongly suggest that the decreased stability of deamidated 4E-BP2 is mediated by increased binding to the Raptor-CUL4B-DDB1 complex.

### Overexpression of Deamidated 4E-BP2 Alters the Neuronal Translational Landscape and Regulates NF-κB Activity

**Figure 5:**
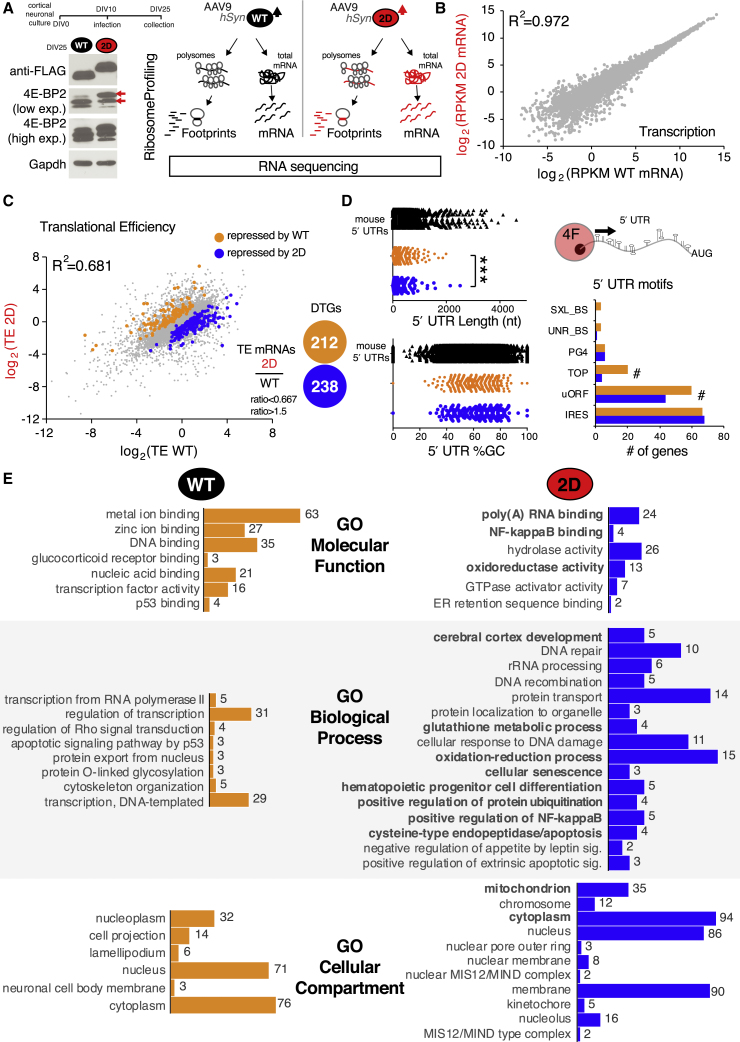
Overexpression of Deamidated 4E-BP2 Alters the Neuronal Translational Landscape (A) Left: overexpression of FLAG-tagged 4E-BP2 (WT or 2D) using AAV9. Representative immunoblots from DIV25 mouse cortical neuron lysates, infected at DIV10. GAPDH is a loading control. Right: diagrammatic depiction of the ribosome profiling experiment. (B) Scatterplot and correlation of RPKM measured from WT or 2D mRNA from DIV25 neurons, as a proxy for transcription, from DIV25 overexpressing neurons. (C) Scatterplot and correlation of translational efficiency (footprint RPKM normalized to mRNA RPKM) between WT and 2D overexpressing DIV25 neurons (log_2_ RPKM of 2D versus WT). Differentially translated genes (DTGs), repressed by WT (orange) or 2D (blue), are shown for 0.667 < ratio > 1.5. (D) 5′ UTR analysis of DTGs versus mouse 5′ UTR collection: length (nt), %GC content, and UTRdb motifs. Data are shown as means ± SEMs. For length and %GC: one-way ANOVA; Bonferroni’s post hoc; ^∗∗∗^p < 0.001; # change in motif abundance. (E) DAVID analysis of DTGs (WT left, orange; 2D right, blue) for Gene Ontology (GO) categories molecular function, biological processes, and CC. The number of genes in each category is shown and the order of categories is by decreasing p value (see Table S3; all p values shown here are <0.05). The categories in bold are discussed further in the text. See also Figure S6 and Tables S3 and S4.

Deamidation of 4E-BP2 during early postnatal brain development could constitute a translational control mechanism targeting repression of a distinct pool of mRNAs. To mimic conditions in which 2D is more abundant due to accumulation, such as following mTORC1 or AMPAR or NMDAR inhibition, we overexpressed 2D in neurons. To map the 2D-regulated translatome, as compared to WT, we carried out unbiased translational profiling using ribosome footprinting coupled with RNA sequencing (Ingolia et al., 2012). DIV10 mouse cortical neurons were infected with adeno-associated virus serotype 9 (AAV9) expressing FLAG-tagged WT or 2D 4E-BP2, driven by the neuron-specific human synapsin (hSyn) promoter (Figure 5A, left). Immunoblotting of neuronal lysates at DIV25 revealed the robust expression of WT and 2D, detected by anti-FLAG antisera (Figure 5A, left). Using a hypotonic lysis buffer, we first extracted polysomes and subsequently isolated ribosome-protected footprints following RNase I nuclease digestion. In parallel, we isolated total RNA from neuronal culture lysates. From both ribosome-protected footprints (a proxy for translation) and total mRNA (a proxy for transcription), we prepared libraries for RNA sequencing (Figure 5A, right). NovaSeq (Illumina) produced high-quality reads for footprint and mRNA libraries, as evidenced first by the r^2^ of reads per kilobase of transcript per million mapped reads (RPKM) between biological replicates, which is >0.9 for both footprints and total mRNA (Figure S6A), second by the canonical distribution of footprint size (28–32 nt) (Figure S6B), third by the read distribution within the 3 frames (Figure S6C), and fourth by the canonical periodicity of ribosomal footprints across mRNA coding and non-coding regions (Figure S6D). RPKM measurements of mRNA libraries demonstrate that there is no significant change in mRNA abundance between WT and 2D as evidenced by R^2^ = 0.972, suggesting that there are no major differences in transcriptional responses (Figure 5B). Conversely, RPKM reads of footprints normalized to mRNA abundance (translational efficiency [TE]) show a pervasive change in the translational landscapes of WT versus 2D 4E-BP2 (R^2^ = 0.681) (Figure 5C). Analysis of log_2_ of TE between 2D and WT replicates (ratio <0.667 and ratio >1.5, respectively; p < 0.05) indicated that 212 genes were upregulated (repressed by WT overexpression), while 238 genes were downregulated (repressed by 2D overexpression), revealing 2 highly dissimilar translatomes (differentially translated genes [DTGs]) for WT and 2D (Figure 5C; Table S3). To elucidate the features of mRNAs, which could render them sensitive to either WT or 2D 4E-BP2 regulation during translation initiation, we obtained 5′ UTR sequences for both lists of targets and carried out length, guanine-cytosine (GC) content, and motif analyses using UTRdb (Grillo et al., 2010). 2D-sensitive mRNA 5′ UTRs were shorter and harbored significantly lfewer terminal oligopyrimidine tract (TOP) and upstream open reading frame (uORF) elements, as compared to WT-sensitive mRNA 5′ UTRs, while %GC content was not different (Figure 5D).

**Figure 6:**
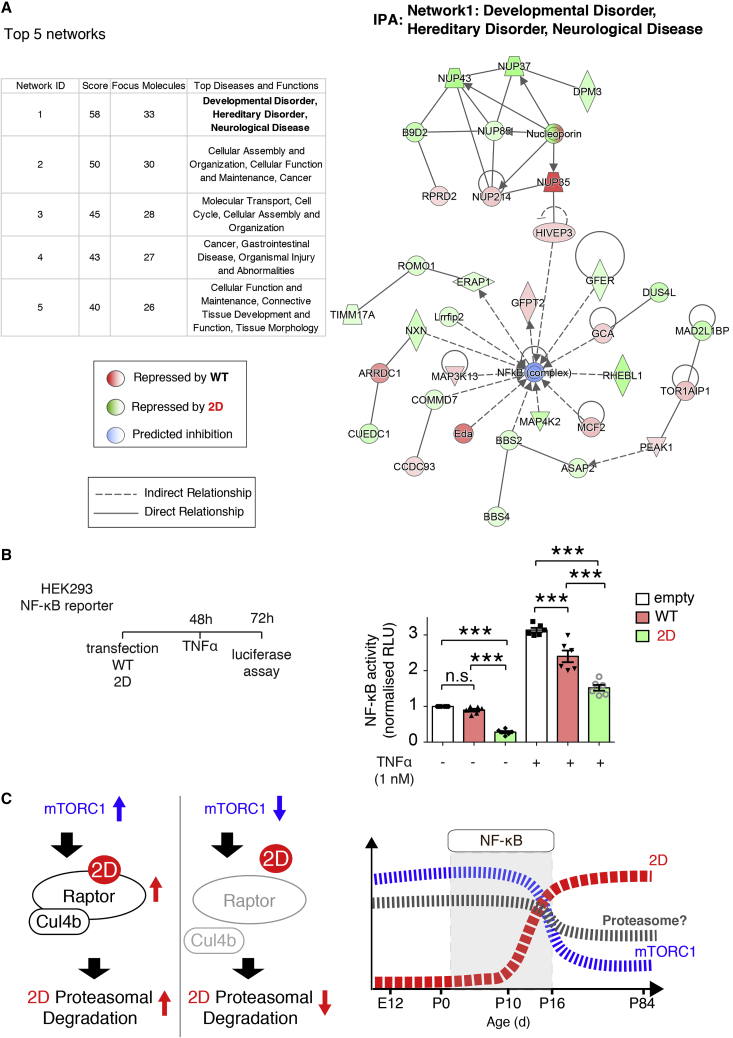
4E-BP2-Mediated Translational Control Regulates NF-κB Activity (A) Left: IPA of ribosome-profiling DTGs from Figure 5 with the molecular activity predictor (MAP) analysis tool. The top five scoring networks are shown. Right: detailed node graph of the top scoring network—developmental disorder, hereditary disorder, and neurological disease—in which NF-κB is the central node. Direct and indirect relations between nodes are shown for DTGs repressed by WT (red) or 2D (green) within the predicted network. (B) NF-κB reporter assay in HEK293 cells using a luciferase-based assay. Two-way ANOVA with Bonferroni’s post hoc; ^∗∗∗^p < 0.001, n = 6 for each group. (C) Diagrammatic summary of the mechanism described in this paper.

Furthermore, to identify pathways affected by 2D-sensitive mRNAs, we carried out Gene Ontology (GO) analysis using DAVID (Figure 5E; Table S4). Multiple GO categories linked to transcription (p < 0.05; biological pathways [BP], molecular function [MF], and cellular compartment [CC]), were identified by DAVID analysis of the 212 WT-repressed genes, suggesting that the overexpression of WT 4E-BP2 could elicit the homeostatic modulation of transcription (Figure 5E, left). In contrast, 2D-repressed genes displayed a DAVID GO profile that was distinct from WT, including categories such as MF: poly(A) RNΑ binding, NF-κ binding; BP: cerebral cortex development, NF-κB activity, glutathione metabolic process, and oxidoreductase activity; and CC: mitochondrion (Figure 5E, right). Moreover, when we carried out ingenuity pathway analysis (IPA) of DTGs using the molecular activity predictor (MAP) tool, we identified networks predicted to be regulated by WT and 2D 4E-BP2 (Figure 6A). The top network is developmental disorder, hereditary disorder, and neurological disease (comprising 21 2D-sensitive genes and 12 WT-sensitive genes), with the central node being NF-κB (Figure 6A). This predicted network suggests that the balance of WT-2D 4E-BP2 is important for regulating NF-κB activity. In summary, deamidated 4E-BP2 represses the translation of a subset of mRNAs, which is distinct from WT 4E-BP2-regulated mRNAs and seems to play a pivotal role in the regulation of NF-κB activity. To test this hypothesis, which originated from our unbiased translatome profiling, we used an NF-κB activity reporter in a stable HEK293 cell line, with a luciferase-based system as a readout (Figure 6B). The overexpression of 2D 4E-BP2 represses both basal and tumor necrosis factor α (TNF-α)-stimulated NF-κB activity, while WT only represses TNF-α-stimulated NF-κB activity (Figure 6B). 2D repression of NF-κB activity following TNFα stimulation is significantly higher when compared to WT (Figure 6B), in agreement with the IPA network prediction (Figure 6A).

Thus, postnatal brain-specific reduction in mTORC1 signaling could regulate brain NF-κB activity by inhibiting proteasome activity and subsequently leading to the accumulation of deamidated 4E-BP2 (Figure 6C).

## Discussion

We describe a previously unidentified mechanism during early postnatal brain development whereby the deamidated form of the cardinal brain translation initiation repressor 4E-BP2 is more susceptible to ubiquitin proteasomal degradation (as compared to non-deamidated protein) because it binds with higher affinity to a complex comprising the mTORC1 protein Raptor and the ubiquitin E3 ligase CUL4B. Deamidated 4E-BP2 stability is regulated by mTORC1 and glutamate receptor activity. The overexpression of deamidated 4E-BP2 represses the translation of a distinct pool of mRNAs linked to cerebral development, mitochondria, and chiefly NF-κB activity.

4E-BP2 deamidation occurs in mouse neurons, but not in glial cells (Figure 1A). Deamidated 4E-BP2 is only detected in postnatal and adult brains, and not in the other peripheral tissues examined (Bidinosti et al., 2010b). Thus, our data support a neuron-centric role for 4E-BP2 deamidation during early postnatal development and into adulthood. Deamidated 4E-BP2 is also present in the human brain (Figure 1B). Potentially, this mechanism is evolutionary conserved from rodents to humans to preferentially regulate neuronal translation during a crucial developmental period for brain growth, synapse regulation, neuronal proliferation and migration, and ultimately, circuitry formation and behavior (Semple et al., 2013, Pressler and Auvin, 2013). The period when deamidation arises (P10–P21) in mice (concomitant with a decrease in mTORC1 activity) would correspond to 10 months–3 years of age in human children (Semple et al., 2013). mTORC1 activity is dysregulated in several monogenic disorders co-diagnosed with high rates of autism, such as fragile X syndrome and tuberous sclerosis (Kelleher and Bear, 2008). Global knockout of 4E-BP2 engenders molecular, cellular, and behavioral phenotypes, which are reminiscent of ASDs (Gkogkas et al., 2013). Other studies have described a key role for 4E-BP2 in synaptic function, learning, and memory (Banko et al., 2005, Banko et al., 2006, Banko et al., 2007, Ran et al., 2013). Given these studies and our work, it is conceivable that dysregulated deamidated 4E-BP2 degradation, as a result of altered mTORC1 signaling during development, could be linked to a prodromal period of neurodevelopmental disorders such as ASDs (via aberrant translational control of neuronal mRNAs).

Postnatal brain-specific asparagine deamidation of 4E-BP2 (N99D/N102D) is spontaneous and pH dependent, and there is no current evidence that it is catalyzed by enzymes (Bidinosti et al., 2010b, Robinson, 2002). However unlikely it may be, an as yet unidentified protein complex or enzyme could carry out 4E-BP2 deamidation, similarly to RIG-I (He et al., 2015), which regulates antiviral cytokine production, or to the immune sensor cGAS (Zhang et al., 2018). Furthermore, the pronounced slower migration of deamidation-corresponding bands on SDS-PAGE suggests that deamidation may affect 4E-BP2 structure (Figure 1). Moreover, mTORC1 phosphorylation induces folding of the intrinsically disordered 4E-BP2 protein (Bah et al., 2015). We did not detect major changes in deamidated 4E-BP2 structure by SAXS, SRCD, or NMR, as compared to WT (Figures 1, S2, and S3); both proteins are IDPs comprising random coils. Structural analysis of deamidated 4E-BP2 in complex with Raptor or eIF4E could reveal undetected conformational changes in its IDP state. WT and 2D subcellular localization was similar, but there was little overlap between the fluorescent puncta corresponding to each form (Figures 1C–1E).

At the same time, for many proteins, deamidation is traditionally viewed as an “aging by-product,” which labels long-lived proteins for degradation (Robinson, 2002). In accordance with this, we do find that 2D is more ubiquitinated and less stable than WT 4E-BP2 (Figure 2). Only the deamidated form and not an alanine mutant (2A) exhibited reduced protein stability, suggesting that this phenotype is specifically induced by the conversion of asparagines to aspartates and not by a non-specific mutation. We then show that 2D stability can be regulated by a major signaling pathway (mTOR) and the activity of glutamate receptors (Figure 3), and that 2D overexpression engenders a widespread alteration of the neuronal translatome (Figure 5), thus demonstrating that 4E-BP2 deamidation is highly regulated by major neuronal signaling pathways and receptor activity, and that it plays a key functional role. The lower stability of 2D, together with the non-overlapping puncta of WT and 2D detected in dendrites (Figure 1D), support the hypothesis that the two forms of 4E-BP2 may regulate the function of different types of synapses, by selective translational de-repression of different mRNAs. In accordance with this premise, 2D was shown to preferentially regulate excitatory AMPA synaptic transmission (Bidinosti et al., 2010b).

Deamidated 4E-BP2 is degraded by binding with higher affinity to the mTORC1 protein Raptor and the ubiquitin E3 ligase CUL4B (Figure 4), while pharmacological inhibition of mTORC1 promotes deamidated 4E-BP2 accumulation (Figure 3) and reduces Raptor-4E-BP2 binding (Schalm et al., 2003). These data reveal that there is a causal relation between Raptor-deamidated 4E-BP2 binding and protein stability under the control of mTORC1 activity. It is plausible that accelerated 2D 4E-BP2 degradation (through the formation of the Raptor-2D-CUL4B complex) could be part of a wider brain-specific mechanism involving CUL4B and the proteasome, mediating translational de-repression downstream of mTORC1 in certain synapses. Regulation of such synapses could be pivotal for the pathogenesis of ASDs and other neurodevelopmental disorders, in which there are known changes in mTORC1 activity (Costa-Mattioli and Monteggia, 2013, Huber et al., 2015, Kelleher and Bear, 2008). We were unable to recapitulate this mechanism for endogenous proteins due to technical challenges with the immunoprecipitation of Raptor or 4E-BP2 in brain tissue and because of the lack of a 4E-BP2 deamidation-specific antibody.

Furthermore, CUL4B is an X-linked intellectual disability (XLID)-associated gene, and its deletion in mice leads to embryonic lethality (Chen et al., 2012, Jiang et al., 2012). In addition, CUL4B overexpression increases ubiquitination and proteasomal degradation of tuberous sclerosis 2 protein (TSC2; an inhibitor of mTORC1 and syndromic ASD gene; Short et al., 1995) and thus promotes mTORC1 signaling (Ghosh et al., 2008). XLID-linked truncating or missense mutations in CUL4B were shown to be defective in promoting the degradation of TSC2 (Wang et al., 2013). Notably, a link between 4E-BP1 and CUL3 was previously shown to promote hypo-phosphorylated 4E-BP1 ubiquitination and degradation, while hyper-phosphorylated 4E-BP1 is refractory to degradation (Yanagiya et al., 2012). Nevertheless, 4E-BP1 is highly expressed in glial cells, while 4E-BP2 (both deamidated and non-deamidated) is predominantly expressed in neurons (Figure 1A), suggesting a potential dichotomy between CUL4B-CUL3 mechanisms in neurons and glia.

Ribosome profiling data highlighted the aspects of a wider brain-specific mechanism involving 4E-BP2 deamidation (Figure 5). The overexpression of 4E-BP2 forms was carried out using AAV constructs driven by hSyn promoters in a culture that is predominantly neuronal (>90% of cells) (Davis and Temple, 1994), while we also demonstrated that deamidation arises in neurons, but not in glia (Figure 1). However, we cannot exclude the possibility that the translational changes detected may be due to a non-neuron autonomous effect on glial cells. Cell-type-specific “translatomics” (e.g., translating ribosome affinity purification [TRAP]; Heiman et al., 2014) would be required to answer this question. We detected a low correlation between the changes in the translatome following overexpression of the two 4E-BP2 forms and no significant changes in transcription (Figures 5B and 5C). Using UTRscan of DTGs, we discovered that WT but not 2D DTGs are enriched in 5′ UTR features previously reported in mTOR- and eIF4E-sensitive mRNAs (Hsieh et al., 2012, Mamane et al., 2007, Thoreen et al., 2012, Truitt et al., 2015) and could result from the reduced binding of 2D to eIF4E (Bidinosti et al., 2010b) and/or from the increased sensitivity of 2D to mTOR inhibition (Figure 3). Consequently, GO functional analysis showed a very small overlap between WT-2D translatomes, revealing that WT overexpression predominantly elicits widespread translational changes in genes involved in transcription, which could constitute a homeostatic response (Figure 5). Conversely, 2D-regulated genes are involved in cerebral cortex development and NF-κB activity (Figure 5).

A main avenue for NF-κB activity regulation perinatally in brain is through de-repression, following phosphorylation of its inhibitor IκBα (nuclear factor of κ light-chain polypeptide gene enhancer in B cells α). This mechanism is brain-derived neurotrophic factor (BDNF) dependent prenatally and BDNF independent postnatally (Gutierrez and Davies, 2011, Gavaldà et al., 2009). Moreover, the top GO CC detected by DAVID in 2D regulated genes is the mitochondrion (Figure 5E). 4E-BPs were shown to regulate mitochondrial dynamics and biogenesis by the translational control of nucleus-encoded mitochondria-related mRNAs (Morita et al., 2013, Morita et al., 2017). We identified 35 mitochondrial mRNAs in 2D-regulated DTGs (Table S3). Remarkably, the highest scoring network predicted by IPA is developmental disorder, hereditary disorder, neurological disease, and NF-κB is the central node (Figure 6A), while we demonstrated that 2D is a stronger inhibitor of NF-κB activity than WT (Figure 6B). It was suggested that synaptic activity promotes a neuronal Warburg effect, shifting neuronal energy metabolism from oxidative phosphorylation toward aerobic glycolysis (Bas-Orth et al., 2017). In parallel, mitochondrial gene expression peaks during synaptogenesis (P0–P21) (Wirtz and Schuelke, 2011). Furthermore, the interplay between NF-κB and the tumor suppressor p53 was proposed to underlie the metabolic switch of the Warburg effect by regulating nuclear and mitochondrial gene expression (Johnson and Perkins, 2012). 4E-BPs were shown to regulate senescence by controlling the synthesis of the p53-stabilizing protein Gas2 (Petroulakis et al., 2009).

Our ribosome profiling data and the existing evidence regarding NF-κB activity and mitochondria emphasize the importance of our proposed brain-specific translational control mechanism of mTORC1- or glutamate receptor-mediated, Raptor-dependent proteasomal degradation of deamidated 4E-BP2 during a critical period of postnatal brain development (Figure 6C), which could go awry in neurodevelopmental disorders such as ASDs.

## STAR★Methods

### Key Resources Table

**Table:** 

REAGENT or RESOURCE	SOURCE	IDENTIFIER
**Antibodies**		

4E-BP2	Cell Signaling Technologies	2845S
4E-BP1 (53H11)	Cell Signaling Technologies	9644S
Phospho-4E-BP1 (Thr37/46) (236B4)	Cell Signaling Technologies	2855S
Phospho-S6 Ribosomal Protein (Ser240/244)	Cell Signaling Technologies	2215S
Ribosomal Protein S6 Antibody (C-8)	Santa Cruz Biotechnology	sc-74459
c-Myc Antibody (9E10)	Santa Cruz Biotechnology	sc-40
Anti-Cullin 4B antibody	Abcam	ab67035
DDB-1	Cell Signaling Technologies	5428S
HA Tag Monoclonal Antibody (2-2.2.14), DyLight 680	ThermoFisher Scientific	26183-D680
Anti-rabbit IgG, HRP-linked Antibody	Cell Signaling Technologies	7074S
HSC 70 Antibody (B-6)	Santa Cruz Biotechnology	sc-7298
DYKDDDDK Tag Monoclonal Antibody (L5), Alexa Fluor 488	ThermoFisher Scientific	MA1-142-A488
Anti-mouse IgG, HRP-linked Antibody #7076	Cell Signaling Technologies	7076S
p44/42 MAPK (Erk1/2) (137F5) Rabbit mAb	Cell Signaling Technologies	4695S
GAPDH (14C10) Rabbit mAb	Cell Signaling Technologies	2118S
Monoclonal Anti-β-Actin antibody produced in mouse	Merck	A5316-100UL
Monoclonal ANTI-FLAG® M2 antibody produced in mouse	Merck	F1804-200UG
Phospho-p44/42 MAPK (Erk1/2) (Thr202/Tyr204) Antibody	Cell Signaling Technologies	9101S
Anti-mouse IgG for IP (HRP)	Abcam	ab131368
Ubiquitin	Cell Signaling Technologies	3933S
*α-*Tubulin	Sigma-Aldrich	T9026
Phospho-Threonine-Proline	Cell Signaling Technologies	9391S
Anti-HA.11 Epitope Tag (Formerly Covance MMS-101R-500)	Cambridge Bioscience	901514
PSD95 (D27E11) XP® Rabbit mAb	Cell Signaling Technologies	3450S
Monoclonal Anti-Glial Fibrillary Acidic Protein (GFAP) antibody produced in mouse - 100UL	Merck	G3893-100UL
Histone H3 (D1H2)	Cell Signaling Technologies	12648S
Synaptophysin - 1	Synaptic Systems	101 011
Raptor (24C12) Rabbit mAb	Cell Signaling Technologies	2280S
His-Tag Antibody	Cell Signaling Technologies	2365S
UBE2L3 Antibody	Cell Signaling Technologies	3848S
MAP2 antibody	Sigma	M9942

**Chemicals**		

TNFα	Sigma	T0157
Cycloheximide	Merck	C7698-1G
Lactacystine	Merck	L6785-.2MG
MG132 (Z-Leu-Leu-Leu-al)	Merck	C2211-5MG
Homoharringtonine	Merck	SML1091-10MG
Torin1	Tocris Bioscience	4247
Rapamycin	LC Laboratories	R5000-100MG
U0126	Tocris Bioscience	1144
Betullinic acid	Merck	B8936
NBQX	Abcam	ab120046
D-AP5	HelloBio	HB0225
Proteasome Substrate III, Fluorogenic, Suc-Leu-Leu-Val-Tyr-AMC	Calbiochem	539142-5MG

**Critical Commercial Assays**		

Dual-Luciferase® Reporter Assay System	Promega	E1910
TriFECTa DsiRNA Kit for hs.Ri.RPTOR.13	IDT	N/A
TriFECTa DsiRNA Kit for hs.Ri.CUL4B.13	IDT	N/A
Clarity Western ECL Substrate	Biorad	1705061
Pierce ECL Western Blotting Substrate	ThermoFisher Scientific	32106
TruSeq Ribo Profile (Mammalian) Kit	Illumina	RPHRM12126
Ribo-Zero Gold (Human/Mouse/Rat) Kit	Illumina	MRZG126
Agilent Small RNA Kit	Agilent Technologies	5067-1549
NEXTflex Small RNA Sequencing Kit v3	Bioo Scientific	NOVA-5132-06
NF-κB reporter (Luc) - HEK293 Recombinant Cell line	BPS Bioscience	60650

**Experimental models: Cell Lines**		

Human Embryonic Kidney cells (HEK293H ATCC® CRL-1573	ThermoFisher Scientific	11631017

**Recombinant DNA**		

pCDNA3-3HA–4E-BP2 WT	Bidinosti et al., 2010b	N/A
pCDNA3-3HA–4E-BP2 N99D/N102D	Bidinosti et al., 2010b	N/A
pCDNA3-3HA–4E-BP2 ΔTOS	Bidinosti et al., 2010b	N/A
pCDNA3-3HA–4E-BP2 N99D/N102D ΔTOS	This paper	N/A
pCDNA3-3HA–4E-BP2 N99A/N102A	Bidinosti et al., 2010b	N/A
pGEX-6P-1-4E–BP2 WT	Bidinosti et al., 2010b	N/A
pGEX-6P-1-4E–BP2 N99D/N102D	Bidinosti et al., 2010b	N/A
Myc-Raptor	Addgene and PMID: 15268862	Cat#: 1859 (discontinued)
AAV9-hSyn1-3XFlag-4E-BP2 WT-IRES-GFP-WPRE	Vector Biolabs	N/A
AAV9-hSyn1-3XFlag-4E-BP2 N99D/N102D-IRES-GFP-WPRE	Vector Biolabs	N/A
His-Ubiquitin	(Hock et al., 2011)	N/A
pTK-RL	(Gkogkas et al., 2008)	N/A

**Software and Algorithms**		

Adobe Illustrator	Adobe	https://www.adobe.com/creativecloud.html
GraphPad PRISM	Graphpad	https://www.graphpad.com/scientific-software/prism/
Fiji ImageJ software	Open source	https://fiji.sc/
Imaris software	Bitplane	https://imaris.oxinst.com/
NIS-Elements-v4.13 software	Nikon	https://www.microscope.healthcare.nikon.com/en_EU
Huygens Software 4.5.1p3	Scientific Volume Imaging	https://svi.nl/HuygensSoftware
ImageStudio Software	LI-COR	https://www.licor.com
ATSAS software suite	(Konarev et al., 2003)	https://www.embl-hamburg.de/biosaxs/software.html
DAMMIN	(Svergun, 1999)	https://www.embl-hamburg.de/biosaxs/dammin.html
GASBOR	(Svergun et al., 2001)	https://www.embl-hamburg.de/biosaxs/gasbor.html
Multifastats	GITHUB opensource code	https://github.com/davidrequena/multifastats
UTRdb/UTRscan	(Grillo et al., 2010).	(Grillo et al., 2010).
Ingenuity Pathway Analysis (IPA)	QIAGEN	https://www.qiagenbioinformatics.com/products/ingenuity-pathway-analysis/
Database for Annotation, Visualization and Integrated Discovery (DAVID)	(Huang et al., 2009)	https://david.ncifcrf.gov/

### Lead Contact and Materials Availability

Further information and requests for resources and unique/stable reagents should be directed to and will be fulfilled by the Lead Contact, Christos G. Gkogkas (christos.gkogkas@ed.ac.uk.) with a completed Materials Transfer Agreement.

### Experimental Model and Subject Details

#### Animals

All procedures were in accordance with UK Home Office and Canadian Council on Animal Care regulations and were approved by the University of Edinburgh and McGill University. C57BL/6J background animals were used (backcrossed for more than 10 generations; pregnant dams to collect E16-18 embryos and P56 males). Food and water were provided *ad libitum*, and mice were kept on a 12 h light/dark cycle. Pups were kept with their dams until weaning at postnatal day 21. After weaning, mice were group housed (maximum of 6 per cage) by sex. Cages were maintained in ventilated racks in temperature (20-21°C) and humidity (∼55%) controlled rooms, on a 12-hour circadian cycle (7am-7pm light period).

#### Human tissue

Post – mortem human brains were acquired from the MRC Edinburgh Brain & Tissue Bank.

See Table S1

#### Cell line cultures

All cell culture reagents were from ThermoFisher Scientific. Human Embryonic Kidney cells (HEK293H ATCC® CRL-1573) were cultured (37°C, 5% CO2) in Dulbecco’s modified Eagle’s medium (DMEM, 11995065) containing 10% fetal bovine serum (10500064) and 1% Pen/Strep (15140148).

#### Primary dissociated cortical neuronal cultures

All reagents for cell culture were from ThermoFisher Scientific unless stated otherwise. E16-18 mouse embryos (male and female) were collected from pregnant dams and cortices were dissected from the brain and immersed in ice cold HBSS solution (14170146) supplemented with 1x Antibiotic/antimycotic mix (15240062) and HEPES solution at concentration 10 mM (15630106). Cells were dissociated after addition of 1 mg/ml Trypsin (LS003702, Lorne Laboratories) and incubation fοr 15 min at 37°C. Then, 0.05 mg/ml Dnase I (D5025-15KU, Merck) was added and the cells were incubated for 5 min at 37°C. After the incubation, Neurobasal media (21103049) was added twice, supplemented with 1x Antibiotic/antimycotic mix, 1x Glutamax (35050038), B-27 (17504044) and 10% Horse Serum (26050088) to inhibit Trypsin. Then, DNase I was added again and the tissue was triturated. The cells were plated on dishes that were coated with 0.05 mg/ml Poly-D-Lysine (P7886, Merck) for 2 h the day before tissue dissection. 5 h after plating, the media was removed and replaced by new media without serum. Half of the media was replaced every 3 days, supplemented with 1 μΜ Cytosine β–D–arabinofuranoside hydrochloride (Ara-C, C6645-25MG, Merck). To obtain glial cultures, Ara-C-free DIV10 neuronal cultures were trypsinised with Trypsin-EDTA (25300054). Cells were washed twice in 1x PBS and replated in DMEM (11995065) supplemented with 10% fetal bovine serum (10500064) and 1% Pen/Strep (15140148).

### Method Details

#### Transfection of cell-lines or primary neurons and reagents

Transfection of HEK293H cells or primary neurons was carried out with Lipofectamine 3000 (L3000008, Thermo Fisher Scientific) in Opti-MEM (31985070, ThermoFisher Scientific) following the manufacturer’s protocol.

#### Protein stability assay

HEK293H cells were transfected with 1-2 μg DNA (or 10 nM siRNA). Pilot experiments were carried out to calculate the required μg for each plasmid construct to ensure equal starting amounts of protein. For protein stability assays, after 48 h, transfected HEK293H cells, (non-transfected cultured neurons or isolated synaptoneurosomes) were treated with 100 μg/ml Cycloheximide (C7698-1G, Merck), 5 μM or 10 μM Lactacystine (L6785-.2MG, Merck), 20 μM MG132 (Z-Leu-Leu-Leu-al, C2211-5MG, Merck), 2 μg/ml HHT (SML1091-10MG, Merck), 250 nM Torin 1 (4247, Tocris Bioscience), 20 nM Rapamycin (R5000-100MG), 20 μM U0126 (1144, Tocris Bioscience), 2.5 μg/ml Betullinic acid (B8936, Merck), 10 μM NBQX (ab120046, Abcam) and 50 μM D-AP5 (HBO225, HelloBio) for the indicated period of time.

#### Adenoassociated viruses (AAV) and infection of cortical cultures

All AAVs were purchased from Vector Biolabs. AAV vectors were cloned by Vector Biolabs: AAV9-hSyn1-3Xflag-4E-BP2 WT-IRES-GFP-WPRE and AAV9-hSyn1-3Xflag-4E-BP2 N99D/N102D-IRES-GFP-WPRE and were used to generate ∼3.5x10^13^ GC/ml for each AAV. Primary dissociated cortical neuronal cultures were infected at DIV10 with 7x10^11^ GC/ml of each virus and collected at DIV25.

#### *In vivo* ubiquitination assay

HEK293H cells were transfected with 5-10 μg of 3xHA-plasmids expressing either WT or 2D 4E-BP2 (pilot experiments were carried out to determine the required amount (μg) for each plasmid construct to ensure equal starting amounts of protein) and 10 μg His-Ubiquitin (Hock et al., 2011). After 48 h of transfection, cells were treated with 20 μM MG132 for 6 h. Cells were lysed in urea buffer (8 M Urea, 0.1 M NaH_2_PO_4_, 0.1 M Tris-HCl [pH 8.0], 0.05% Tween 20, and 10 mM imidazole [pH 8.0]). 5 mg of total protein was incubated with Ni-NTA Agarose beads (30210, QIAGEN) overnight to pull down ubiquitinated proteins. The beads were washed twice with denaturing wash buffer (8 M Urea, 0.1 M NaH_2_PO_4_, 0.1 M Tris-HCl [pH 8.0], 0.05% Tween 20, 20 mM imidazole [pH 8.0]) and then with native wash buffer (0.1 M NaH_2_PO_4_, 0.1 M Tris-HCl [pH 8.0]. Protein was dissolved in Laemmli buffer and resolved by SDS-PAGE. Monoclonal antibody HA.11 (901514, Cambridge Bioscience) was used to detect ubiquitinated 4E-BP2.

#### *In vitro* ubiquitination assay

*In vitro* ubiquitination assay was performed in 100 μL reaction mixture at 37°C for 2 h. The reaction mixture included 100 ng purified human recombinant 4E-BP2 WT and N99D/N102D, 100 ng purified human recombinant UBE1 (E1 enzyme, E-304, BostonBiochem), 500 ng UbcH7/UBE2L3 (E2 enzyme, E2-640, BostonBiochem), 10 μg ubiquitin (U-530, BostonBiochem), 2.5 μg purified human recombinant CUL4B (E3 enzyme, H00008450-P01, Novus Biologicals), 50 ng purified human recombinant DDB1 (ab114333, abcam), purified human recombinant Raptor (H00057521-P01, Novus Biologicals) in an ATP-regenerating system [50 mM Tris-HCl, pH 7.6, 10 mM MgCl_2_, 2 mM ATP (R0441, ThermoFisher Scientific) 10 mM creatine phosphate (10621714001, Merck), 3.5 U/mL creatine kinase (10127566001, Merck) and 0.6 U/mL inorganic pyrophosphatase (M0361S, New England Biolabs)], in the presence of 5 μM ubiquitin aldehyde (U-201, BostonBiochem) and 50 μM MG132. Proteins were dissolved in Laemmli buffer and resolved by SDS-PAGE.

#### Proteasome activity assay

The chymotrypsin-like activity of the proteasome was determined using a specific proteasome substrate (Proteasome Substrate III, Fluorogenic, Suc-Leu-Leu-Val-Tyr-AMC, 539142-5MG, Calbiochem). Synaptoneurosome fractions (10 μg) were incubated with the substrate (40 μM) in 100 μL of proteasome assay buffer [0.05 M Tris-HCl (pH 8.0), 0.5 mM EDTA, 1 mM ATP, and 1 mM dithiothreitol (DTT)] at 37°C for 1 h. After the incubation, proteasome activity was measured every 20 min and the plate was kept at 37°C. The fluorescence of the released AMC was detected using a fluorescence microplate reader system (GloMax Explorer Multimode Microplate Reader, Promega) at 380-nm excitation and 460-nm emission wavelengths.

#### NF-κB reporter luciferase assay

The NF-κB reporter (luc)-HEK293 cell line (BPS Bioscience) contains a firefly luciferase gene driven by four copies of NF-κB response element located upstream of the minimal TATA promoter. Stable cells were transfected with 4E-BP2 isoforms as above and supplemented with 100 ng pTK-RL (Gkogkas et al., 2008) for 48h and lysed 24h later (+/− TNFα 1nm) in lysis buffer from the Dual Luciferase Reporter Assay kit (Promega). Firefly and renilla luminescence were measured using a FLUOstar OPTIMA micro-plate reader (BMG LABTECH). Firefly luciferase luminescence values were normalized to renilla firefly luminescence values and are averages of four experiments.

#### Immunoblotting

HEK293H cells, dissociated cortical neuronal cultures or mouse/human tissue were lysed in RIPA buffer (150 mM sodium chloride, 1.0% NP-40, 0.5% sodium deoxycholate, 0.1% SDS, 50 mM Tris, pH 8.0) supplemented with protease and phosphatase inhibitors (Roche) unless otherwise specified, in a Dounce glass homogenizer by applying ∼30 strokes, on ice. Samples were further incubated on ice for 15 min, with occasional vortexing, and cleared by centrifugation for 20 min at 16,000 x *g* at 4°C. The supernatant was used for western blotting after the protein concentration of each sample was determined by measuring A_280_ absorbance on a NanoDrop (ThermoFisher Scientific). 50 μg of protein per lane was prepared in Laemmli sample buffer (50 mM Tris, pH 6.8, 100 mM DTT, 2% SDS, 10% glycerol, 0.1% bromophenol blue), heated to 98°C for 2 min, and resolved on 10%–16% polyacrylamide gels. Proteins were transferred to 0.2 μm nitrocellulose membrane (Bio-Rad), blocked in 5% milk in TBS-T (10mM Tris, pH 7.6, 150mM NaCl, 0.1% Tween20) for 1 h at room temperature, incubated with primary antibodies 1:1000 (1% BSA in TBS-T containing 0.02% Na azide) overnight at 4°C and with secondary antibodies 1:5000 for 1 h at room temperature (5% milk in TBS-T). Between incubations, membranes were washed three times in TBS-T. For reprobing, membranes were stripped by incubation with 0.2 M NaOH for 10 min and blocked with 5% milk in TBS-T for 1 h. Proteins were visualized using enhanced chemiluminescence (1705061, Biorad and 32106, ThermoFisher Scientific) after exposing on X-ray films (34089, ThermoFisher Scientific) processed with an Ecomax Film Processor (ProTec).

#### Immunoprecipitation

HEK293H cells were transfected with 5 μg DNA of the HA plasmids expressing either WT or 2D (the amount of DNA was balanced to achieve the same intensity/protein expression for each plasmid) and 10 μg of Myc – Raptor. After 48 h of transfection, cells were rapidly homogenized in ice cold lysis buffer (50mM HEPES pH 7.5, 1% CHAPS, 150mM NaCl, protease and phosphatase inhibitors), on ice. Homogenates were incubated at 4°C with constant rotation and centrifuged at 15,000 x *g* for 10 min at 4°C. Supernatants were collected and precleared with 100 μL of protein G agarose beads (37478S, Cell Signaling Technologies). 7 mg of precleared supernatant was incubated with 3 μg of c-*myc* antibody [(9E10), sc-40, Santa Cruz] for 30 min at 4°C, followed by incubation with protein G agarose beads overnight at 4°C. Beads were then centrifuged at 3,500 x *g* for 1 min at 4°C and washed three times with lysis buffer for 10 min. Immunoprecipitates were dissolved in 2X Laemmli buffer, resolved by SDS-PAGE and probed with anti-myc and anti-HA antisera.

#### Phosphatase Treatment

Whole brains or cortical neuronal cells at the indicated ages were homogenized in 1X phosphatase buffer (PMP Buffer, B0761S, New England Biolabs) containing protease inhibitors (Roche), supplemented with 1 mM MnCl_2_ (B1761S, New England Biolabs). Extracts were diluted to 2 μg/μl in a total volume of 90 μl. 9 μL of the phosphatase (P0753S, New England Biolabs) was added per sample and the samples were incubated at 30°C for 45 min. The reactions were stopped by addition of 5X SDS-PAGE Laemmli sample buffer.

#### Isolation of Synaptoneurosomes

Synaptoneurosomes were prepared from fresh mouse brain tissue. Cortices were isolated from WT mice aged 8-12 weeks, and each hemisphere was homogenized in ice–cold sucrose buffer (320mM Sucrose, 5mM Tris, 1mM EDTA, pH 7.4) and the homogenates were centrifuged for 10 min, at 1000 x *g*, 4°C. The supernatant was kept on ice and the pellet was resuspended in sucrose buffer and centrifuged again for 10 min, 1000 x *g* at 4°C. The pooled supernatant was then centrifuged for 10 min, at 21,000 x *g* at 4°C to pellet out crude synaptoneurosomes. Crude synaptoneurosomes were resuspended in 3% Percoll (GE Healthcare) and layered on a discontinuous 10%–24% Percoll gradient. The material between layers 24% and 10% was collected, resuspended in Ionic Media (20mM HEPES, 10 mM Glucose, 1.2mM Na_2_HPO4, 1 mM MgCl_2_, 5mM NaHCO3, 5mM KCl, 140mM NaCl, pH 7.4) and centrifuged at 21,000 x *g*, for 15 min at 4°C.

#### Immunofluorescence and Confocal Imaging

Primary cortical neuronal cultures were prepared from E17 mouse embryos. Cells were plated on coverslips, previously coated with 0.05 mg/ml poly-D-lysine (P7886, Merck) for 2 h and 10 μg/ml laminin (23017-015, Invitrogen) for 1 h, at a density of 80,000 cells/well in 24-well dishes. Four days after plating, neurons were co-transfected with 0.25 μg of HA - 4E-BP2 WT plasmid and 0.25 μg of FLAG – 4E-BP2 N99D/N102D using 0.5 μL of Lipofectamine 3000 in pre-warmed Opti-MEM supplemented with 1x Glutamax. Following 1 h of transfection, neurons were returned to conditioned media. Neurons were fixed at DIV16 in 4% PFA in phosphate-buffered saline (PBS) for 8 min and washed three times for 5 min in PBS. Cells were permeabilised with 0.1% Triton X-100 for 5 min and blocked with 2.5% BSA in 1x PBS for 30 min. Then, cells were incubated with 1:50 anti-FLAG Tag Monoclonal Antibody (L5), Alexa Fluor 488 (MA1-142-A488, ThermoFisher Scientific) and 1:25 anti-HA Tag Monoclonal Antibody (2-2.2.14), DyLight 680 (26183-D680, Thermo Fisher Scientific) for 2 h. Coverslips were incubated with DAPI 1:10000 (4’,6-Diamidino-2-Phenylindole, Dihydrochloride, D1306, Thermo Fisher Scientific) for 5 min. Then, the coverslips were washed and mounted with Lab Vision PermaFluor Aqueous Mounting Medium (TA-030-FM, Thermo Fisher Scientific). Images of co-transfected neurons were acquired on a Nikon A1R microscope using a 60X objective. For the quantification and colocalization analysis experiments, z stack images were taken with a pixel size of 60 × 60 nm^2^ and z-step size of 150 nm. Excitation laser wavelengths for the different samples were: 488 nm for FLAG tag, 680 nm for HA tag, and 401.5 nm for DAPI. Microscope control and image acquisition were done using the NIS-Elements-v4.13 software.

#### Imaging analysis

Deconvolution of confocal images was performed using Huygens Essential (Huygens Software 4.5.1p3) before subsequent analysis. Co-localization analysis was performed on 3D, deconvolved images and quantified using ImarisColoc (Imaris v8.2.1, Bitplane Inc, software available at https://imaris.oxinst.com). All image analysis and quantification of images acquired with confocal microscopy were performed on deconvolved images without any additional processing. Brightness and contrast settings were adjusted in Imaris for presentation purposes only.

#### Ribosome profiling

Components of the Epicenter TruSeq Ribo Profile (Mammalian) Kit (Illumina, RPHRM12126), with some modifications, were used to generate sequencing libraries. In brief, polysomes were extracted from primary neuronal cultures infected with 4E-BP2-expressing viruses. A partial volume of these lysates was digested with TruSeq Ribo Profile Nuclease (RNase I) (Ribosome Protected Fragments, RPF), while another part of the lysate was kept as an internal transcription control (Total mRNA). After digestion, RPFs were purified on MicroSpin S-400 columns as described in the kit to enrich for small RNA fragments (28-30 nt).

All samples (RPF and Total mRNA) were depleted of ribosomal RNA using the Ribo-Zero Gold (Human/Mouse/Rat) Kit (Illumina, MRZG126). RPFs only were purified on a 15% TBE-Urea polyacrylamide gel (EC68852BOX, ThermoFisher Scientific), selecting bands running between 28 and 30 nt. Only Total RNA samples were heat fragmented. All samples were end-repaired using TruSeq Ribo Profile Polynucleotide kinase. Samples were quantified using the Agilent Small RNA Kit (Agilent Technologies). For library generation, NEXTflex Small RNA Sequencing Kit v3 for Illumina Platforms (Bioo Scientific) was used. Input was balanced between samples to ensure similar output. The manufacturer’s protocol was followed. In brief, an adenylated 3′ adaptor was ligated, followed by an adenylated 5′ adaptor ligation. The RNA fragments were then reverse transcribed into cDNA and amplified using PCR (18 cycles). During the PCR, individual samples were barcoded for multiplex sequencing using the barcoding primers compatible with Illumina sequencing, included in the kit. The PCR products were size selected on an 8% native TBE-PAGE gel (EC62152BOX, ThermoFisher) and purified from the gel according to the manufacturer’s instructions. The cDNA libraries were then analyzed for size, quantity and quality using the Agilent High Sensitivity DNA kit (Agilent Technologies).

Samples were balanced and pooled for sequencing with Edinburgh Genomics on NovaSeq S1 flow cells yielding 50 bp paired-end reads. Raw sequencing data were de-multiplexed by the sequencing facility. Sequences were analyzed using a custom developed bioinformatics pipeline adapted from Ingolia et al. (2012) and previously described in Amorim et al. (2018). Adapters were removed from raw sequencing reads using FASTX Toolkit and undesired rRNA and tRNA sequences were removed via alignment using bowtie. Filtered reads were mapped to an indexed reference genome using STAR and raw counts per gene obtained from the aligned data.

Raw counts were converted to Reads Per Kilobase of transcript per Million mapped reads (RPKM) for each gene *i* in RPF and total RNA using the formula:

$$RPKM_{i}=\frac{count_{i}}{\frac{length_{i}}{10^{3}}*\frac{\sum_{i=1}^{k}count_{i}}{10^{6}}}$$

For each gene, the translational efficiency (TE) was calculated by dividing the RPKM values of the RPF libraries by the RPKM values of the total RNA libraries. The R package Xtail (Xiao et al., 2016) was used for formal testing of the TE ratio significance in pairwise comparisons of treatments. Changes in transcription (total RNA) between predefined pairwise comparisons of treatments were analyzed using methods of microarray normalization as described in Amorim et al. (2018). False-discovery rates (FDR) were calculated from p values derived with the z-score as in Reiner et al. (2003). Genes with < 40 reads were discarded. Raw, aligned RNaseq data will be deposited to NCBI Gene Expression Omnibus (GEO) and are currently available from the following link: https://datasync.ed.ac.uk/index.php/s/AvexXZSretGYl2X and the password is: deamidation

#### UTR analysis

UTRs were obtained from Biomart ENSEMBL (Yates et al., 2016) using the GRCm38.p6 version of the mouse genome. 5′ UTR motifs were predicted using UTRscan, pooling data from UTRdb (Grillo et al., 2010). Data Length in BP and %GC content were calculated using free Python-based scripts (Multifastats; https://github.com/davidrequena/multifastats).

#### Gene Ontology and Pathway Analysis

Gene Ontology (GO) and Pathway Analysis were performed using, respectively, the online tool DAVID version 6.8 (Huang et al., 2009), and the Ingenuity Pathway Analysis Software (IPA; QIAGEN; version 42012434). Differentially translated genes were submitted to IPA and subjected to Core Analysis with analysis parameters set to include Direct and Indirect Interactions and Experimentally Observed data only. Network data were obtained for all datasets and Molecular Activity Predictor (MAP) analysis was applied based on the differentially regulated genes belonging to each individual network. For GO analysis, filtered gene lists set to highlight genes differentially repressed by WT or 2D were individually submitted to DAVID and GO annotation gathered for Biological Function, Molecular Function and Cellular Component.

#### Protein expression and purification

Recombinant proteins were expressed in *Escherichia coli* BL21(DE3) (C600003, ThermoFisher) by growing transformed cells in LB medium at 37°C, inducing with 1 mM isopropyl β-D-1 thiogalactopyranoside (IPTG, I6758, Merck). After 3 h of induction at 28°C, the cells were harvested, washed with 100 mM Tris-HCl (pH 7.5), 170 mM NaCl and lysed in 20 mM PBS, (pH 7.4), 270 mM NaCl, 5 mM KCl, 1 mM DTT, 0.1 mg/ml lysozyme (L6876, Merck) by one freeze-thaw cycle, followed by sonication. Lysed cells were centrifuged at 16,000 x *g* for 30 min at 4°C and the supernatant was loaded onto a glutathione-Sepharose resin (GE17-0756-01, GE Healthcare), allowing the protein to bind for 4 h at 4°C. The resin was then washed with 10mM PBS, (pH 7.4), 140 mM NaCl, 3 mM KCl and 1 mM DTT. Recombinant His-tagged 3C protease was added to the resin and incubated for 18 h at 4°C. Tagless protein was collected and loaded onto a Ni-NTA resin (30230, QIAGEN) to bind the 3C protease. Tagless protein was eluted with 20 mM imidazole. Eluted fractions were loaded onto a Superdex S200 16/600 column (GE Healthcare), equilibrated with 20 mM Tris-HCl pH 7.4 and 150 mM NaCl. 4E-BP2 gave one monodisperse peak, which was collected and 1 mM DTT was added. Protein samples were then concentrated using a 3 kDa MWCO spin concentrator to 4-11 mg/ml. Protein concentration was determined by absorbance measurements at 280 nm and the protein identity was confirmed by tryptic in-gel digestion and mass spectrometry using the method described previously (Raasakka et al., 2015).

Protein samples used for NMR were prepared as stated above but expressed in M9 minimal medium supplied with N^15^ labeled ammonium chloride. The buffer used in the final SEC purification step consisted of 20 mM PBS pH 7.4 and 150 mM NaCl.

#### Size exclusion chromatography - multi angle light scattering (SEC-MALS)

The molecular mass of 4E-BP2 was determined with SEC-MALS, using a miniDAWN Treos MALS detector (Wyatt). Protein concentration was measured with an online RI detector. The SEC column, Superdex S200 Increase 10/300 (GE Healthcare), was equilibrated with 20 mM Tris-HCl (pH 7.4), 150 mM NaCl at 4°C. The SEC-MALS system was calibrated using ovalbumin, and the concentration of the injected 4E-bP2 was 1.2 mg/ml.

#### Synchrotron radiation circular dichroism (SRCD)

Recombinant purified proteins were either diluted into a buffer (20 mM PBS pH 7.4, 150 mM NaF and 0.5 mM DTT) just before the measurement or dialyzed against 20mM phosphate pH 7.5 for 20 h at 4°C. The ellipticity of each sample was measured between 170 and 280 nm in a quartz cuvette with a pathlength of 0.1 mm on the AU-CD beamline at ASTRID2 (ISA, Aarhus, Denmark) at 10°C. Sample concentrations were between 0.3-1.0 mg/ml and the same protein concentration was used, when different samples were compared.

#### Small angle X-ray scattering (SAXS)

SAXS data were collected on the BM29 beamline (Pernot et al., 2013) of the European synchrotron radiation facility (ESRF, Grenoble, France), using a wavelength of 0.9919 Å. For batch measurements, 20 frames were collected with 0.5 s of exposure per frame at a temperature of 10°C. Sample concentrations were within a range of 0.5-10 mg/ml. SEC-SAXS was also performed using an Agilent BioSEC-3 HPLC column, equilibrated with 20 mM Tris-HCl (pH 7.4), 150 mM NaCl, collecting one frame/s. Data were processed and analyzed using the ATSAS software suite (Konarev et al., 2003). The radius of gyration was calculated either based on the Guinier region or using the Debye formula (Calmettes et al., 1994). 3D *ab initio* models were generated using DAMMIN (Svergun, 1999) and GASBOR (Svergun et al., 2001).

#### Nuclear magnetic resonance spectroscopy (NMR)

Purified ^15^N labeled proteins (9 mg/ml) were measured in a buffer consisting of 20 mM PBS (pH 7.4), 150 mM NaCl and 1 mM DTT with 10% D_2_O and 2,2-dimethyl-2-silapentane-5-sulfonate (0.1 mM), using an 850 MHz Bruker BioSpin 850 Ascend sepctrometer at 27°C.

### Quantification and Statistical Analysis

#### Quantification of Immunoblotting

The intensity of each protein band was measured from original images (no brightness or contrast adjustments) with ImageStudio Software (Li-COR Biosciences) in triplicate and averaged to minimize measuring variability. Loading controls were used in each experiment. Data are shown as protein expression (arbitrary units) after normalization to control. For quantification of endogenous 4E-BP2 in brain, the intensity of the bottom band was measured for WT 4E-BP2 and the intensity of middle and top band corresponding to single or double deamidated 4E-BP2 respectively, for 2D 4E-BP2.

#### Statistical Analysis and Experimental Design

Experimenters were blinded to the genotype during experimentation and data analysis. All data are presented as mean ± SEM (error bars) and individual experimental points are depicted in column or bar graphs. Statistical significance was set *a priori* at 0.05 (n.s.: non-significant). Where analysis of variance (ANOVA) is carried out the assumptions for normality (Shapiro-Wilk) and equality of variances (Bartlett’s test) are met. No nested data were obtained in this study; we only collected one observation per research object. The n number denotes biological replicates. No randomization was carried out for any of the experiments described here. Details for statistical and post hoc tests used were provided within figure legends or the relative methods description and summarized in Table S2; all data collected followed normal distributions, thus only parametric tests were used. Data summaries and statistical analysis were carried out using Graphpad Prism 6 unless otherwise stated.

### Data and Code Availability

Original/raw data will be available upon reasonable request from the Lead Contact for academic/non-commercial purposes. Ribosome profiling sequencing data will be available from Mendeley (https://doi.org/10.17632/2dmgbfht62.1).

## Supplemental Information

Document S1. Figures S1–S6 and Table S1

Table S2. Statistical Analysis Details, Related to All Figures

Table S3. DTGs from Ribosome Profiling, Related to Figure 5

Table S4. DAVID GO Analysis Summary, Related to Figure 6

Document S2. Article plus Supplemental Information
