# Supplementary material for: Raptor-Mediated Proteasomal Degradation of Deamidated 4E-BP2 Regulates Postnatal Neuronal Translation and NF-κB Activity
Source: Cell Rep. 2019 Dec 10;29(11):3620–3635.e7. doi: 10.1016/j.celrep.2019.11.023 (PMC6915327; doi:10.1016/j.celrep.2019.11.023)
Supplement: Document S1. Figures S1–S6 and Table S1 [file mmc1.pdf]

Cell Reports, Volume *29*

Supplemental Information

Raptor-Mediated Proteasomal Degradation of Deamidated 4E-BP2 Regulates Postnatal Neuronal Translation and NF-κB Activity

Stella Kouloulia, Erik I. Hallin, Konstanze Simbriger, Inês S. Amorim, Gilliard Lach, Theoklitos Amvrosiadis, Kleanthi Chalkiadaki, Agniete Kampaite, Vinh Tai Truong, Mehdi Hooshmandi, Seyed Mehdi Jafarnejad, Paul Skehel, Petri Kursula, Arkady Khoutorsky, and Christos G. Gkogkas

Supplemental Information

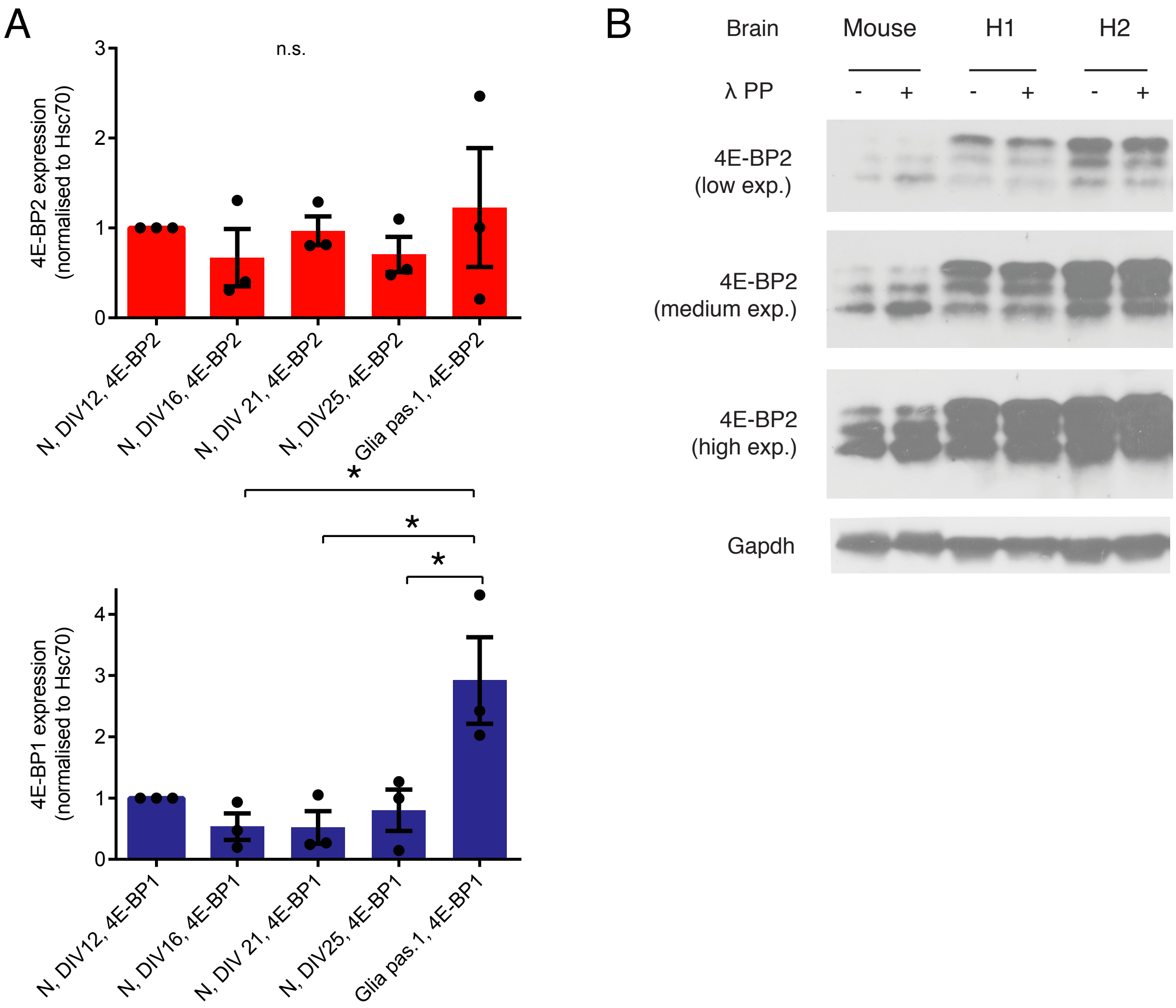

Figure S1 4E-BP2 deamidation in human brain - Related to Fig. 1. See Tables S1, S2**. A.** Quantification of 4E-BP expression detected with 4E-BP1 or 4E-BP2 specific antisera; One-way ANOVA n=3; *p<0.05. **B.** Representative immunoblot of lysates from one mouse and two post-mortem human brains (H1, H2) treated with λ PPase and probed with antisera against the indicated proteins.

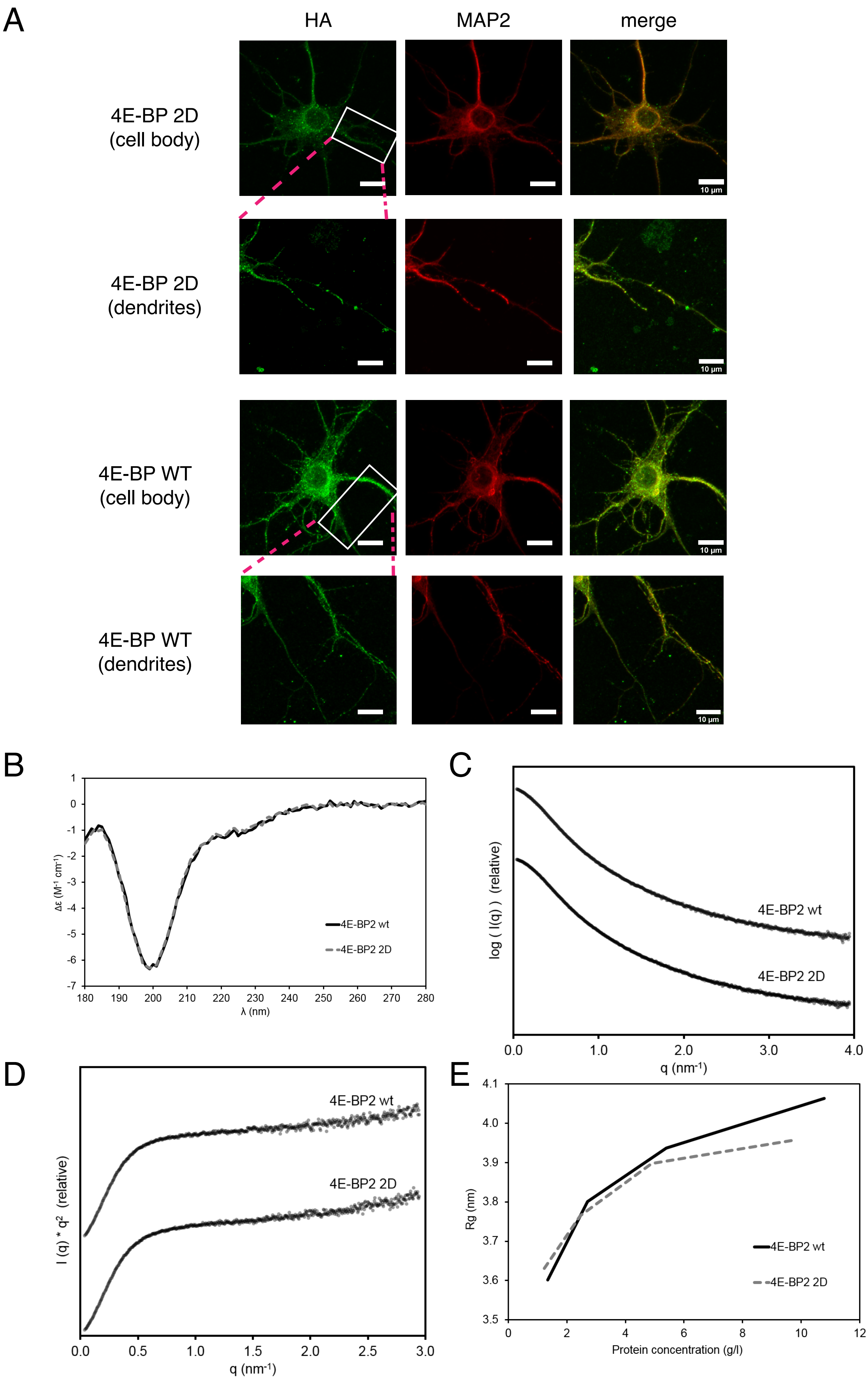
A
HA
MAP2
merge
4E-BP 2D
(cell body)
4E-BP 2D
(dendrites)
4E-BP WT
(cell body)
4E-BP WT
(dendrites)
10 µm
B
Δε (M⁻¹ cm⁻¹)
λ (nm)
4E-BP2 wt
4E-BP2 2D
C
log (I(q)) (relative)
q (nm⁻¹)
4E-BP2 wt
4E-BP2 2D
D
I (q) * q² (relative)
q (nm⁻¹)
4E-BP2 wt
4E-BP2 2D
E
Rg (nm)
Protein concentration (g/l)
4E-BP2 wt
4E-BP2 2D

Figure S2 4E-BP2 localises in dendrites & WT and 2D 4E-BP2 structures consist of random coils and are intrinsically disordered - Related to Fig. 1**. A.** Representative confocal images from immunofluorescent staining of DIV18 cortical neurons transfected with the indicated 4E-BP2 constructs and stained with anti-HA and anti-MAP2. White boxes denote the dendritic area that was magnified. Single and merged images (red/green channels are shown). Biophysical data on the 4E-BPs including **B.** SRCD spectra, **C.** SAXS scatter profiles, **D**. Kratky plots, **E.** Rg calculated by the Debye function plotted versus the protein concentration during SAXS measurements.

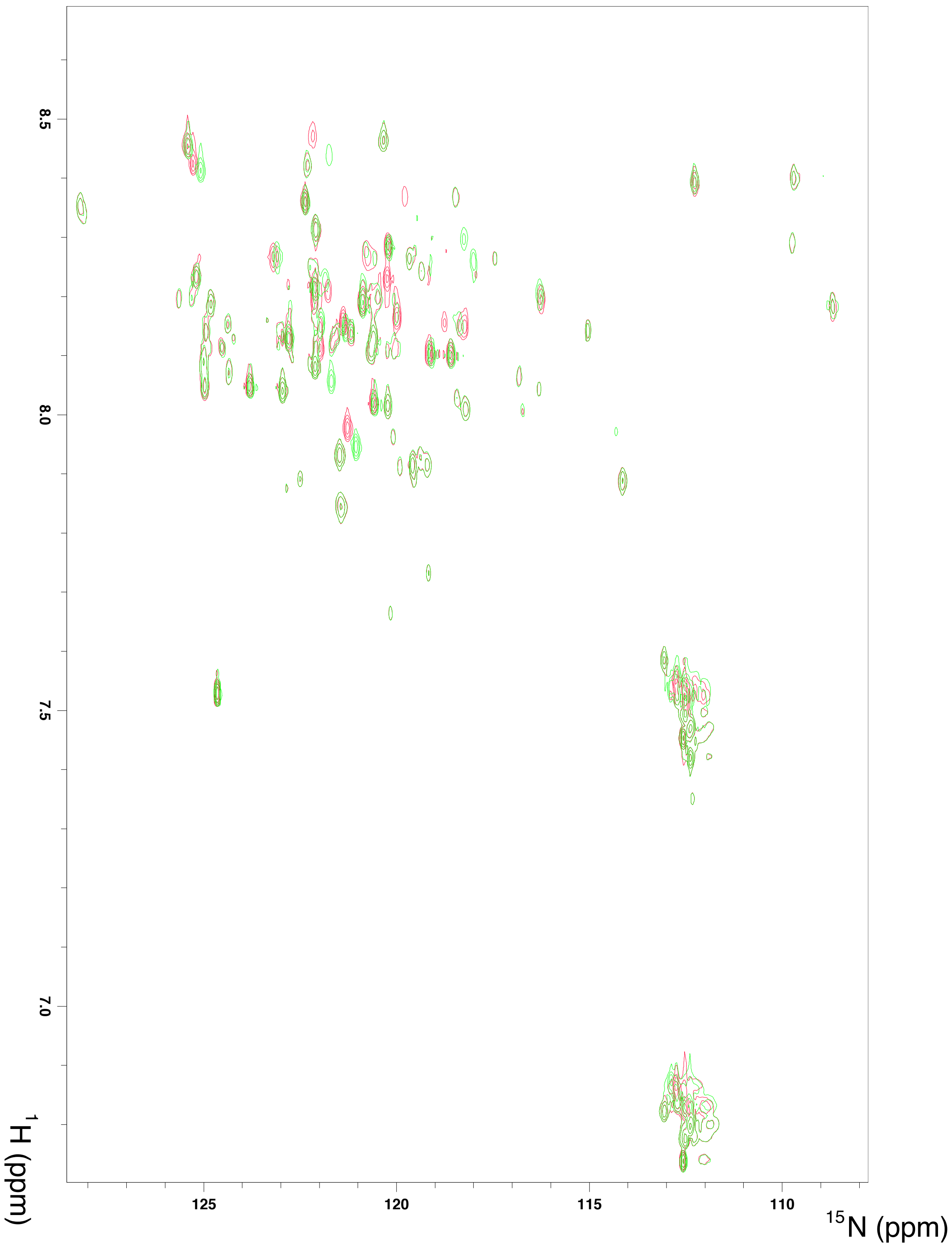

Figure S3 ^{1}H-^{15}N HSQC spectra for purified recombinant WT (green) and 2D (red) 4E-BP2 protein - Related to Fig. 1**.** x-axis: ω_1 - ^{15}N (ppm); y-axis: ω_2 - ^{1}H (ppm).

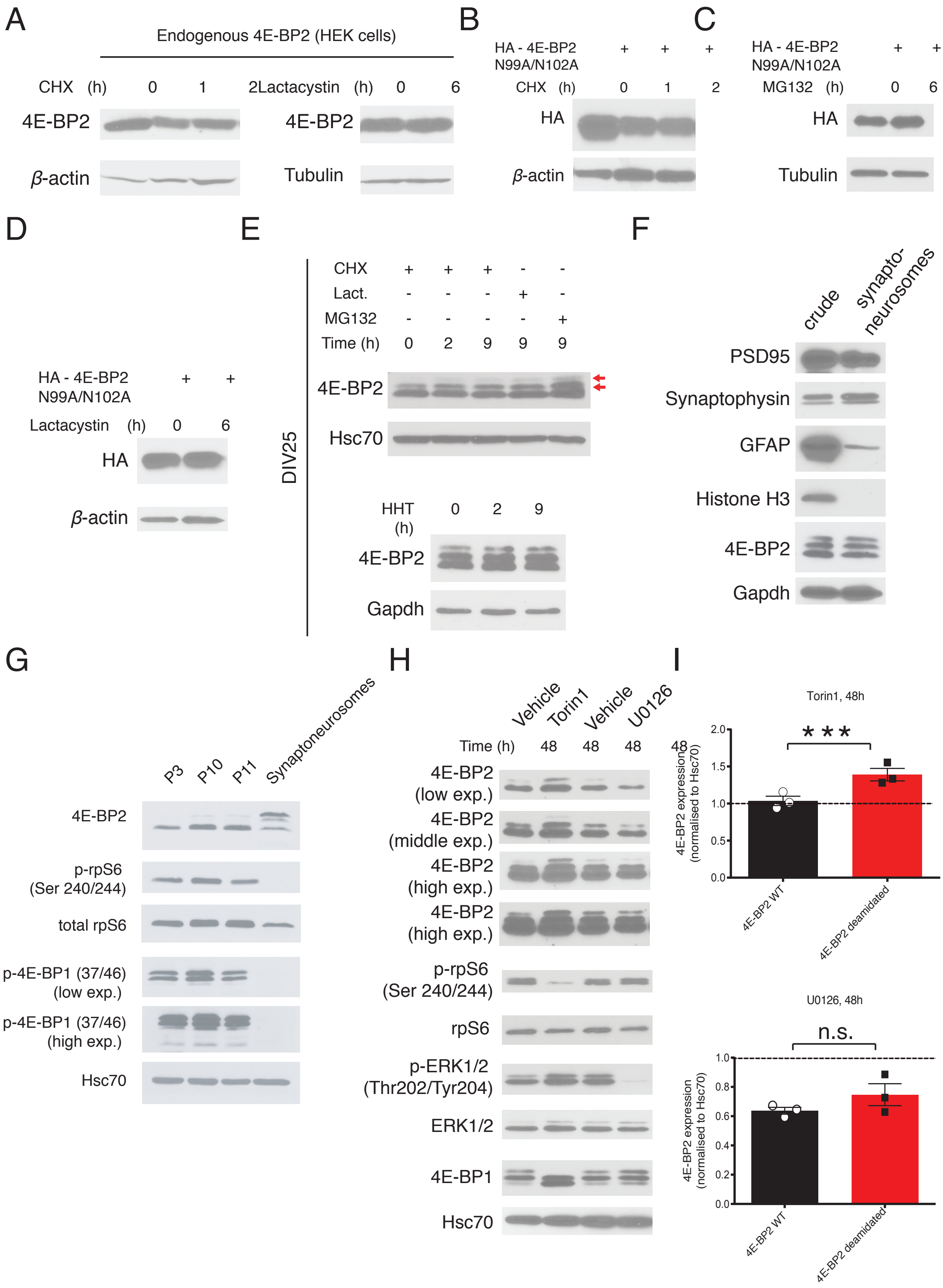

Figure S4 Stability assays of HEK-293H endogenous 4E-BP2 and of a N99A/N102A, of neuronal endogenous 4E-BP2; quality control of synaptoneurosomes; mTORC1 activity in glia; Torin 1 48h treatment - Related to Fig. 2, 3. See Table S2**. A., B., C. and D.** Protein stability assays in HEK-293H

cells for endogenous 4E-BP2 and transfected N99A/N102A mutant. Representative immunoblots of lysates treated with: A: CHX, Lact, B: CHX, Cycloheximide (CHX) 100 μg/ml; 0, 1 and 2 h, Lactacystin (Lact.) 5 μM; 0, 6 h, **C.** MG132 20 μM; 0, 6 h and **D.** Lact. Membranes in A, B, C, D probed with antisera against the indicated proteins. **E.** Protein stability assays in DIV25 dissociated mouse cortical neurons. Representative immunoblots of lysates treated with: for DIV25; Cycloheximide 100 μg/ml; 0, 2 and 9 h (CHX), MG132 20 μM; 0, 9 h, Lactacystin (Lact.) 5 μM; 0, 9 h and Homoharrintonine (HHT) 2 μg/ml; 0, 9 h; probed with antisera against the indicated proteins. **F.** Representative immunoblots from P56 mouse brain extracted synaptoneurosomes and crude lysates probed with antisera against the indicated proteins. For A, B C, D, E, F: α-tubulin, β-actin, Gapdh or Hsc70 are loading controls. **G.** Representative immunoblot from P3, P10 and P11 cultured glia lysates from mouse cortex (synaptoneurosomes lysate is also shown). Lysates were probed with antisera against the indicated proteins**. H.** Representative immunoblots from cultured neurons treated with vehicle or 250 nM Torin 1; for 48 h, probed with antisera against the indicated proteins. **I.** Quantification of immunoblots from H; 4E-BP2 expression normalised to control is shown for non-deamidated and deamidated 4E-BP2. For I: Data are shown as mean ± S.E.M. (error bars); n=3 per condition; The intensity of the vehicle band is set as 1 (dotted line on graph). Two-way ANOVA; Bonferroni's post-hoc; $*p<0.05$.

For A, B, C, D, E, F: n=3.

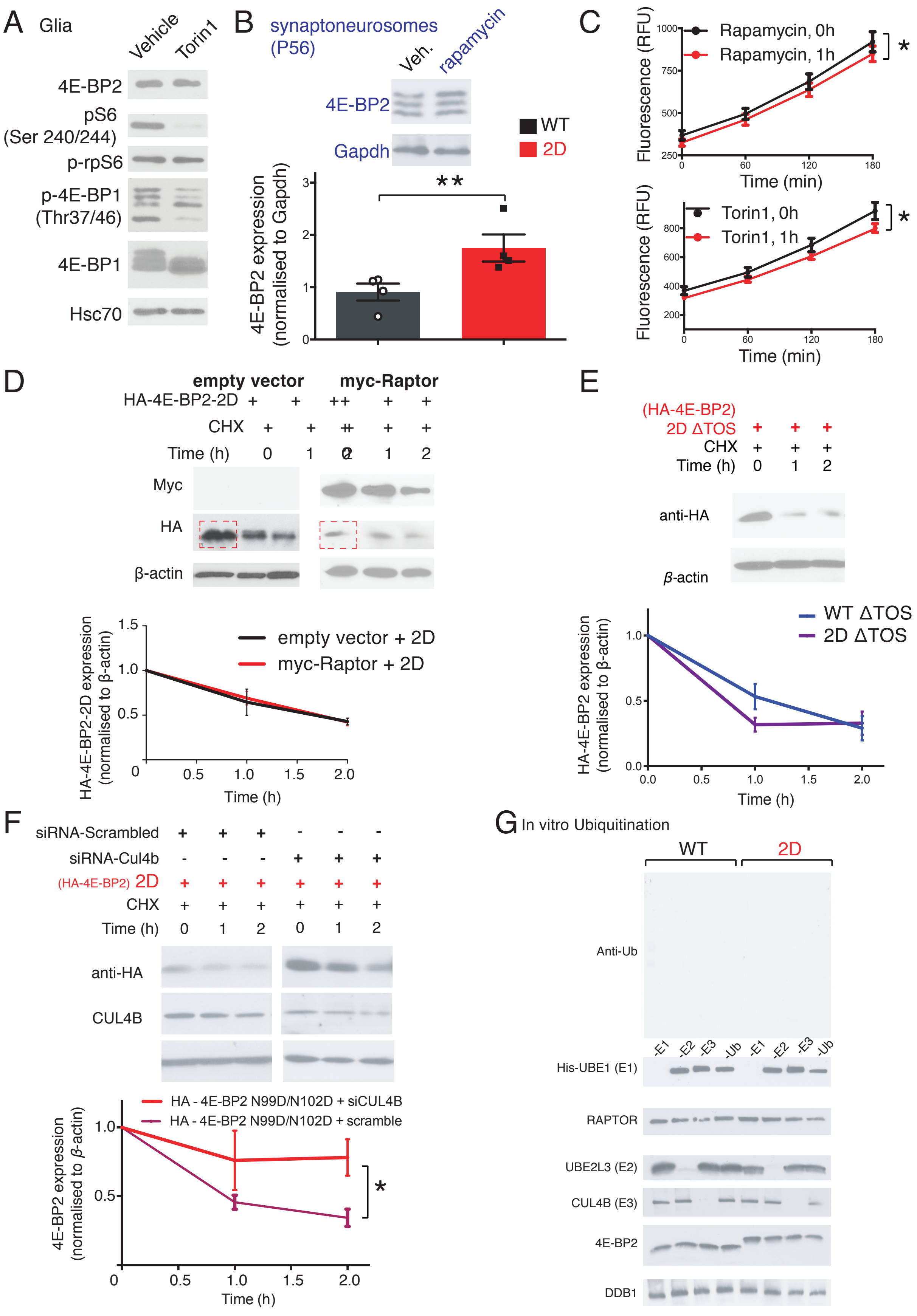
A Glia
Vehicle
Torin1
4E-BP2
pS6 (Ser 240/244)
p-rpS6
p-4E-BP1 (Thr37/46)
4E-BP1
Hsc70
B synaptoneurosomes (P56)
Veh.
rapamycin
4E-BP2
Gapdh
WT
2D
4E-BP2 expression (normalised to Gapdh)
**
C
Rapamycin, 0h
Rapamycin, 1h
Fluorescence (RFU)
Time (min)
Torin1, 0h
Torin1, 1h
*
D
empty vector
myc-Raptor
HA-4E-BP2-2D
CHX
Time (h)
Myc
HA
β-actin
empty vector + 2D
myc-Raptor + 2D
HA-4E-BP2-2D expression (normalised to β-actin)
Time (h)
E
(HA-4E-BP2) 2D ΔTOS
CHX
Time (h)
anti-HA
β-actin
WT ΔTOS
2D ΔTOS
HA-4E-BP2 expression (normalised to β-actin)
Time (h)
F
siRNA-Scrambled
siRNA-Cul4b
(HA-4E-BP2) 2D
CHX
Time (h)
anti-HA
CUL4B
HA - 4E-BP2 N99D/N102D + siCUL4B
HA - 4E-BP2 N99D/N102D + scramble
4E-BP2 expression (normalised to β-actin)
Time (h)
*
G In vitro Ubiquitination
WT
2D
Anti-Ub
-E1
-E2
-E3
-Ub
His-UBE1 (E1)
RAPTOR
UBE2L3 (E2)
CUL4B (E3)
4E-BP2
DDB1

Figure S5 mTORC1 inhibition does not affect 4E-BP2 stability in glia; the effect of rapamycin treatment on 4E-BP2 stability in synaptoneurosomes; ΔTOS constructs stability assays in HEK293H cells; CUL4B-dependent degradation of 4E-BP2- Related to Fig. 3, 4**. A.** Representative immunoblots from glia passage 1 (p.1) treated with vehicle or 250 nM Torin 1; for 48 h, probed with antisera against the indicated proteins; n=3. Hsc70 is the loading control. **B.** Top: Representative immunoblots from P56 mouse brain extracted synaptoneurosomes treated with vehicle or 20 nM Rapamycin; for 1 h, probed with antisera against the indicated proteins; Bottom: Quantification of immunoblots from; 4E-BP2 expression normalised to control is shown for wild-type (WT) and deamidated (2D) 4E-BP2. Data are shown as mean ± S.E.M. (error bars); n=4 per condition; The intensity of the vehicle band is set as 1 (dotted line on graph). Two-way ANOVA; Bonferroni's post-hoc; *p<0.05. **C.** Proteasome enzymatic activity assays in treated synaptoneurosomes with 250 nM Torin1 or 20 nM Rapamycin – corresponds to samples analysed by western blotting in Fig. 3D and Fig. S5B. Proteasome activity is shown in RFU/min ± S.E.M. (n = 4) (see table S2 for statistical analysis). **D.** Representative immunoblots from HEK-293H lysates co-transfected with empty vector or myc-Raptor and 2D HA-tagged 4E-BP2. The experiment was carried out in the presence of 100 μg/ml cycloheximide (CHX) for 0, 1 or 2 h; *β*-actin is the loading control. Bottom: Quantification of HA-expression measured by immunoblotting, normalised to *β*-actin. Red square indicates ~70% decrease in HA expression. **E.** Representative immunoblots from HEK-293H lysates co-transfected with HA-tagged 2D or 2D ΔTOS. Experiment was carried out in the presence of 100 μg/ml cycloheximide (CHX) for 0, 1 or 2 h; *β*-actin is the loading control. Bottom: Quantification of HA-expression (corresponding to WT or 2D) measured by immunoblotting, normalised to *β*-actin **F.** Top: Representative immunoblots from HEK-293H lysates co-transfected with siRNA (scrambled or against *CUL4B)* and 2D HA-tagged 4E-BP2. The experiment was carried out in the presence of 100 μg/ml cycloheximide (CHX) for 0, 1 or 2 h; *β*-actin is the loading control. Bottom: Quantification of HA-expression (corresponding to WT or 2D) measured by immunoblotting, normalised to *β*-actin. For B, C, D, E, F data are shown as mean ± S.E.M. (error bars); n=3 per condition; For B *Student's t*-test. For C, D, E, F Two-way ANOVA; Bonferroni's post-hoc; **p<0.01, *p<0.05. **G.** In vitro ubiquitination assay control reactions using purified GST-4E-BP2 WT or 2D. All reactions were performed in the presence of purified Raptor and DDB1 proteins and probed with antisera against the indicated proteins.

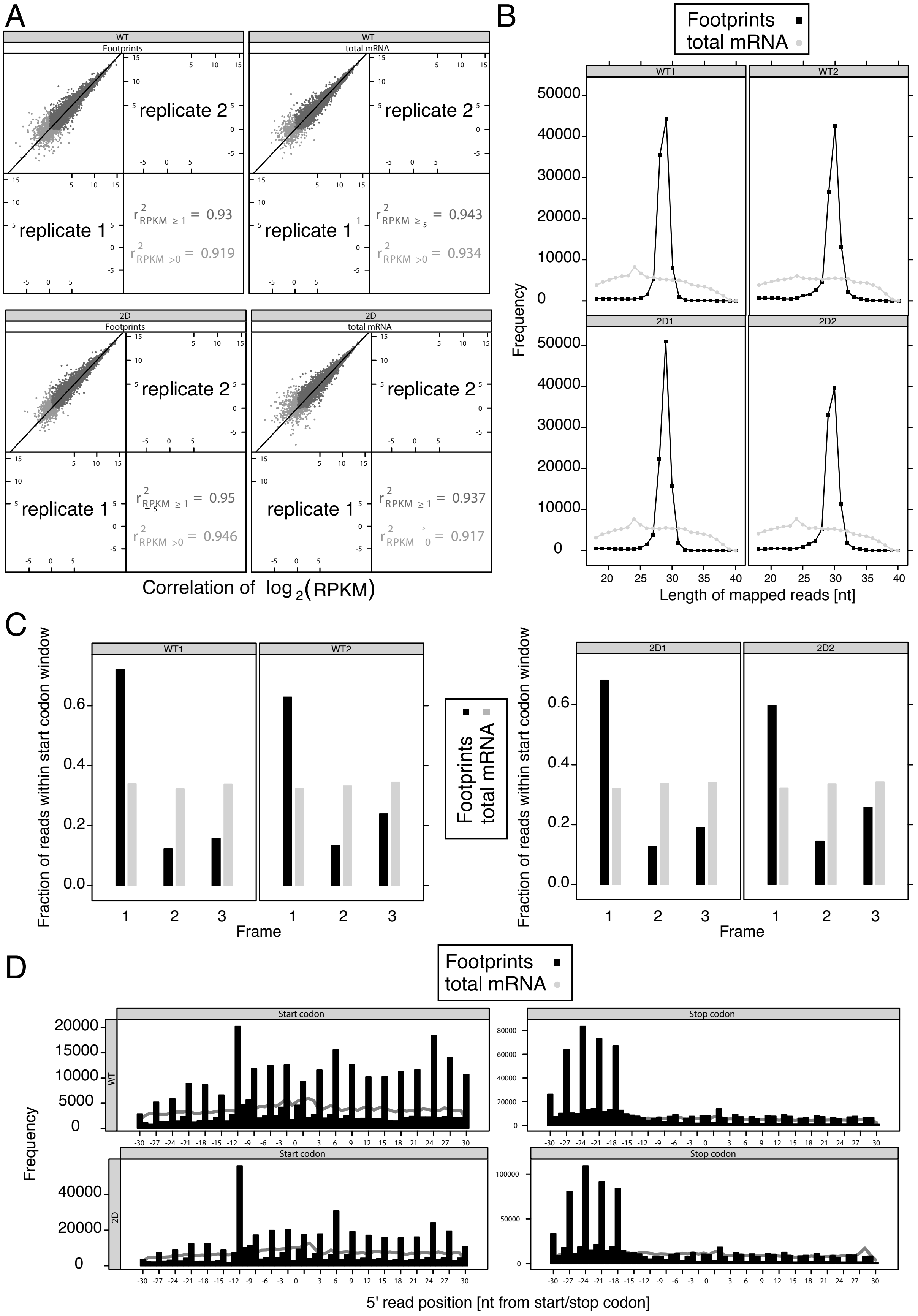

Figure S6 Reproducibility and QC of ribosome profiling experiment - Related to Fig. 5**. A.** Reproducibility plots for WT and 2D sequenced libraries [for replicates of total mRNA and footprints

(light grey corresponds to data points with <40 reads)]. **B.** Frequency and length of mapped reads and **C.** Cumulative reading frame usage for total mRNA and footprint libraries (WT and 2D). **D.** Frequency of reads for footprint (black; showing the expected 3nt periodicity) and total mRNA libraries (grey) in relation to 5′ read position (start and stop codon shown).

ID	Sex	Age	Area	MRC BRAIN BANK NUMBER
SD038/15	Male	44	Posterior Cyngulate Gyrus BA23	BBN 26313
SD034/15	Male	69	Posterior Cyngulate Gyrus BA23	BBN 26308

Post – mortem human brains were provided from the MRC Edinburgh Brain & Tissue Bank.

Table S1 Anonymised human Post-mortem brain tissue information - Related to Figure 1.
